# Germline haploinsufficiency of *MUTYH* causes mutational signature SBS18 in multiple tumour types and specifically raises colorectal cancer risk

**DOI:** 10.1038/s41698-026-01425-x

**Published:** 2026-05-06

**Authors:** Kitty Sherwood, Juan Fernandez-Tajes, Güler Gül, Steve Thorn, Joseph C. Ward, James F. Wilson, Claire Palles, D. Timothy Bishop, Richard S. Houlston, Malcolm G. Dunlop, Ian P. M. Tomlinson

**Affiliations:** 1 Department of Oncology, Old Road Campus Research Building, University of Oxford, Oxford, UK; 2 Cancer Research UK Scotland Centre, Institute of Genetics and Cancer, University of Edinburgh, Edinburgh, UK; 3 MRC Human Genetics Unit, University of Edinburgh, Edinburgh, UK; 4 Centre for Genomic and Experimental Medicine, Institute of Genetics and Cancer, University of Edinburgh, Edinburgh, UK; 5 Centre for Global Health Research, Usher Institute, University of Edinburgh, Edinburgh, UK; 6 Institute of Cancer and Genomic Sciences, College of Medical and Dental Science, University of Birmingham, Birmingham, UK; 7 Leeds Institute of Medical Research, University of Leeds, Leeds, UK; 8 Division of Genetics and Epidemiology, The Institute of Cancer Research, Sutton, UK

**Keywords:** Cancer, Genetics, Oncology

## Abstract

The MUTYH base excision repair protein corrects oxidative DNA damage. Bi-allelic germline *MUTYH* mutations cause a rare, Mendelian recessive syndrome of colorectal adenomas, duodenal polyps and colorectal cancer (CRC), in which tumours have excess somatic C:G > A:T mutations and the mutational signature SBS36. Signature SBS18, which resembles SBS36, is common in sporadic CRCs and other cancers. Increased risks of CRC and other cancers have been reported in germline *MUTYH* heterozygotes (mono-allelic mutation carriers, frequency 2-3%), but the existence of these associations and underlying mechanism have remained controversial. Compared with *MUTYH-*wildtype individuals, CRCs from *MUTYH* heterozygotes had ~2.5-fold excess of signature SBS18, increased C:G > A:T transversions (including in driver genes) and raised mutation burden. These observations resulted from *MUTYH* haploinsufficiency, rather than somatic loss of the wildtype allele, contrary to previous suggestions. Hypermutation probably begins before cancer initiation. In a case-control analysis, we found approximately 1.4-fold elevated risk of CRC in *MUTYH* heterozygotes, causally mediated through increased SBS18. The association between *MUTYH* heterozygosity and SBS18 was also present in many extra-colonic cancers, including other gastrointestinal tumours, but the raised SBS18 did not detectably increase the risk of these cancers. Germline heterozygotes for another base excision repair gene, *MBD4*, showed equivalent associations for SBS1, C:G > T:A mutations and CRC risk. Mutational signatures in cancers can result in part from non-rare germline variation in DNA repair. The specific effect of *MUTYH* heterozygosity on CRC risk plausibly reflects the high baseline levels of oxidative damage and SBS18 activity in the colorectum.

## Introduction

Mendelian cancer syndromes frequently show recessive inheritance, implying that a single functional copy of the gene can provide enough residual function to prevent tumorigenesis. Affected individuals generally carry bi-allelic pathogenic germline variants, and may be homozygous for a single variant or compound heterozygotes, with two different pathogenic variants in trans. Most recessive cancer syndromes take the form of DNA repair deficiency and/or a tendency to hypermutation^1^. The underlying defects can include a failure to repair chromosomal-scale lesions (e.g. Fanconi anaemia), environmentally-induced lesions (e.g. xeroderma pigmentosum), DNA replication errors (e.g. constitutional mismatch repair deficiency) and ‘spontaneous’ mutations (e.g. deamination of methylcytosine). An ongoing question that applies to all of these syndromes is whether mono-allelic mutation carriers (often simply referred to as ‘heterozygotes’) are also at increased cancer risk who are evidently much more common than bi-allelic mutation carriers. This is clearly the case for genes such as *BRCA2*^2^, the Lynch syndrome loci^3^ and *ATM*^4^, for which heterozygotes are prone to specific tumour types that overlap partially with those present in the recessive syndrome^5^. Although a haploinsufficiency mechanism is theoretically possible, the increased risk of cancer in carriers of these mono-allelic mutations generally occurs through spontaneous somatic ‘second hits’, in classical tumour suppressor gene fashion^6^.

Given that somatic second hits are a proven mechanism of increased cancer risk, it is reasonable to ask whether all mono-allelic carriers of mutations associated with recessive DNA repair deficiency syndrome alleles have a raised risk of cancer, since spontaneous somatic inactivation of the germline wildtype allele can occur through spontaneous loss of heterozygosity or another mechanism. However, strong statistical support for an increased cancer risk in heterozygotes is often hard to obtain: risk allele frequencies can be as low as 0.1% or less; family-based studies are prone to ascertainment bias; and apparent germline heterozygotes may actually carry second, cryptic germline mutations. Irrespective of the question of raised cancer risk in DNA repair deficiency heterozygotes *via* the two-hit model, even chance second hits at these loci could, in principle, create null DNA repair genotypes and hence specific, important vulnerabilities. Therapeutic strategies against other DNA repair components, or to promote DNA damage of a specific type, could thus provide a cancer cell-specific reduction in viability. However, supporting evidence for ‘second hits’ in tumours is often lacking, because tumour samples are either unavailable or not analysed fully, or only a limited genomic assessment could be performed.

The *MUTYH* (mutY homologue) gene maps to chromosome 1p34.1 and encodes a protein with an important role in the base excision repair (BER) of oxidative DNA damage, removing adenine paired with 8-oxo-guanine, and thus preventing C:G > A:T base substitutions^7^. Approximately 0.05% of the population have bi-allelic, pathogenic germline *MUTYH* variants that cause a constitutional deficiency in the repair of oxidative damage^8^. In European populations, the majority of such individuals are homozygous or compound heterozygous for two missense *MUTYH* variants, p.Gly396Asp and p.Tyr179Cys (ENST00000710952.2), which have individual allele frequencies of about 0.1–1.0% and encode a protein deficient in correcting 8-oxoG:A replication mispairs^9^. Carriers of bi-allelic germline *MUTYH* mutations are predisposed to a high-penetrance recessive syndrome, MUTYH-associated polyposis (MAP), that comprises multiple colorectal adenomas and colorectal carcinomas (CRCs), with a smaller risk of duodenal adenomas and carcinomas. The tumours display mutational signature SBS36^10–12^, which is typified by TCA > TAA and TCA > TAT substitutions, and sometimes the similar signature SBS18, which is found in many sporadic CRCs and is thought to arise from oxidative DNA damage in general (Supplementary Fig. 1). Tumorigenesis in MAP largely occurs along conventional genetic pathways, *via* an increased burden of somatic C:G > A:T changes in driver genes such as *APC, KRAS* and *SMAD4*^13^. An increased risk of other cancer types in MAP remains unproven.

There is a longstanding, unresolved controversy as to whether the 2–3% of the population who are germline *MUTYH* heterozygotes (mono-allelic mutation carriers) have an increased risk of CRC and/or other cancers^14–17^. Studies addressing this question have differed in their sizes, genotyping methods, patient inclusion criteria and their matching of cases and controls, and there is the risk of accidental inclusion of undetected bi-allelic mutation carriers. In one such recent study, Barreiro et al.^16^ found an overall excess of cancer in *MUTYH* heterozygotes, driven principally by patients with adrenocortical carcinoma, oesophageal carcinoma, sarcoma, prostate adenocarcinoma and renal clear cell carcinoma (but not CRC). Mechanistically, somatic loss of the wildtype *MUTYH* allele was postulated to lead to loss of MUTYH activity and thus to hypermutation, raised SBS18 or SBS36 activity, and accelerated tumorigenesis.

Here, we analyse the features of germline *MUTYH* heterozygotes in the UK 100,000 Genomes Project (100kGP), including their cancer risk and the molecular characteristics of their cancers. We then address similar factors for another BER gene *MBD4*. Our results help to elucidate the magnitude and mechanism of cancer risk in BER gene germline heterozygotes, and indicate that COSMIC mutational signatures can originate from germline variation in DNA repair that is not rare (allele frequency ≥1%).

## Results

### Germline and somatic *MUTYH* genotypes in 100kGP CRCs

**Fig. 1:**
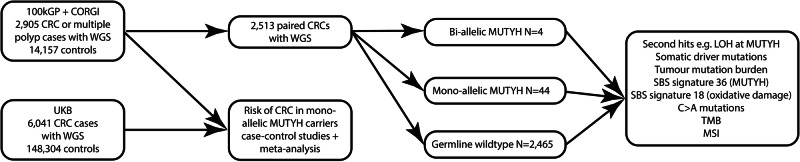
Study design. Consort-style diagram of CRC patients included in germline and somatic genetic analyses.

**Fig. 2:**
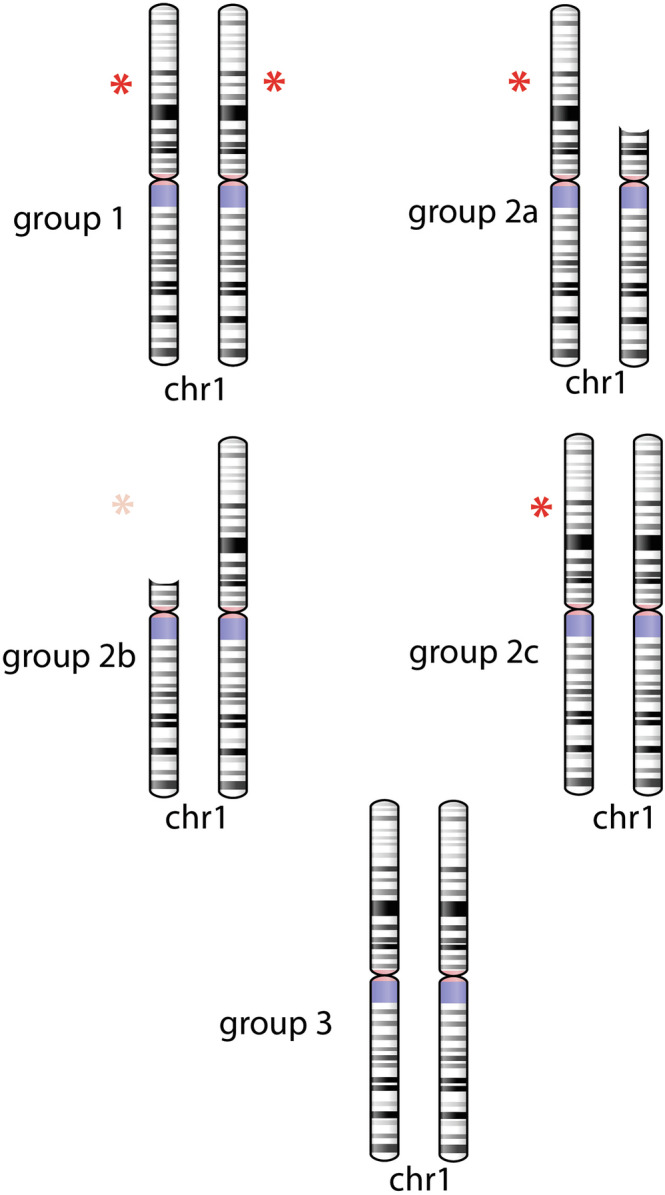
Chromosome 1 ideograms showing the group 1, 2a, 2b, 2c and 3 genotypes in cancers. Germline *MUTYH* mutations are shown by red stars, and somatic second hits by LOH are shown as removing part of the chromosome short arm (copy-loss LOH), although in reality they may instead simply render part of the chromosome homozygous (copy-neutral LOH) in some tumours.

**Fig. 3:**
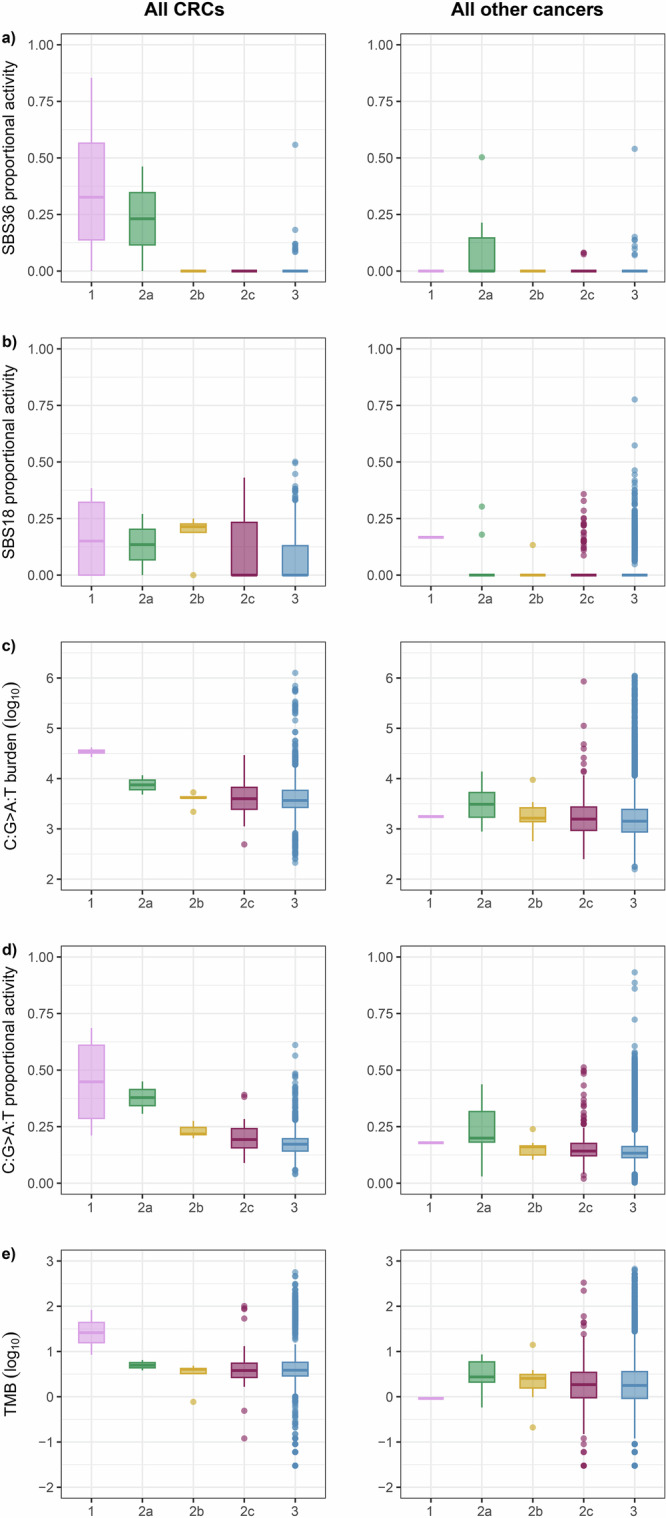
Selected somatic molecular features of tumours in the 100kGP CRC analysis in relation to germline *MUTYH* status. Group 1, bi-allelic germline mutations; 2a, heterozygous with somatic loss of germline wildtype; 2b, heterozygous with somatic loss of germline mutant; 2c, heterozygous with no somatic mutation; and 3, germline wildtype. **a** SBS36 proportional activity, **b** SBS18 proportional activity, **c** C:G > A:T burden, **d** C:G > A:T proportional activity and **e** tumour mutational burden (measured as SCVPM) in germline *MUTYH* genotype in 100kGP CRCs (left) and All Other Cancers (right). Log scales are used to aid display in some cases. Significant pairwise univariable associations at *P* < 0.05 in Wilcoxon tests were, in each panel: **a** group 1 vs groups 2 and 3, **b** group 2 vs group 3, **c** group 1 vs groups 2 and 3 and **d** both group 1 vs groups 2 and 3, and group 2 vs group 3. Details of these associations from Fisher’s exact, *χ*^2^, Wilcoxon and regression analyses are shown in full in Supplementary Tables 4 and 5.

We examined the tumour genomes of 2513 CRCs that we analysed as part of the 100kGP (Fig. 1)^18^. We classified patients into five groups, defined by their germline and somatic *MUTYH* status (Figs. 1–3, Supplementary Tables 1 and 2):

- bi-allelic germline mutations (*n* = 4);
- mono-allelic germline mutation with somatic LOH of the wildtype allele (*n* = 2);
- mono-allelic germline mutation with somatic LOH of the mutant allele (*n* = 5);
- mono-allelic germline mutation with no somatic LOH (*n* = 37); and
- wildtype germline (*n* = 2465).

Somatic mutational processes in tumours were measured in three related ways: (1) *Prevalence* denoted the presence or absence of a mutational signature (e.g. prevalence of SBS36 measured the proportion of cancers with any SBS36 activity); (2) *Burden* measured the absolute numbers of a mutation type in a cancer, such as a specific single base change or mutations assigned to a particular signature; and (3) *Proportional activity* assessed the proportion of all mutations of a specific type (e.g. SBS36 activity was the percentage of mutations assigned to SBS36, relative to all mutations assigned to an SBS signature in that cancer). Prevalence was used to show whether a specific signature was active within a tumour, burden was particularly useful for identifying hypermutant tumours, and activity corrected for background burdens to identify the most active processes in a single tumour. Further details are provided in ‘Methods’. No tumour had a germline *MUTYH* mutation accompanied by a somatic mutation other than LOH, and there was no evidence of somatic *MUTYH* hypermethylation in a subset of 328 CRCs with EPIC v2 850 K CpG methylation data (details not shown).

Groups 2a, 2b and the much larger 2c had similar C:G > A:T, SBS36 and SBS18 metrics (*P* > 0.15, Kruskal-Wallis test**;** Table 1, Supplementary Tables 1 and 2) and were therefore combined into a single group 2 for subsequent analyses, with the exception of specific investigations of the effects of somatic loss of the germline wildtype *MUTYH* allele (group 2a).

**Table 1:** Synopsis of associations between germline *MUTYH* genotypes and somatic mutation metrics in 100kGP cancers

MUTYH groups	Somatic mutation metric	CRC only				CRC-excluded				Stat. Test
		Mean 2a vs. 2b/c	Median 2a vs. 2b/c	*P*	*n*	Mean 2a vs. 2b/c	Median 2a vs. 2b/c	*P*	*n*	
Mono-allelic with LOH mutant vs. othermono-allelic groups 2a vs. 2b/c	Presence SBS36	0.50 vs. 0		0.091	2 group 2a vs. 42 group 2bc	0.36 vs. 0.01		1.42 × 10^−4^	10 group 2a vs. 211 groups 2bc	F
	Burden SBS36	5999 vs. 0	5999 vs. 0	7.70 × 10^−6^		1244 vs. 7	0 vs. 0	**3.30** **×** **10**^**−12**^		W
	Activity SBS36	0.231 vs 0	0.231 vs. 0	7.70 × 10^−6^		0.091 vs. 0.001	0.000 vs. 0.000	**3.30** **×** **10**^**−12**^		W
	Presence SBS18	0.50 vs. 0.50		1.00		0.27 vs. 0.13		0.77		F
	Burden SBS18	2117 vs. 2781	2117 vs. 805	0.90		1176 vs. 537	0 vs. 0	0.51		W
	Activity SBS18	0.135 vs. 0.122	0.135 vs. 0.072	0.81		0.059 vs. 0.024	0.000 vs. 0.000	0.52		W
	Burden C>A	8249 vs. 6417	8249 vs. 4162	0.25		3979 vs. 7536	2597 vs. 1569	0.040		W
	Activity C>A	0.379 vs. 0.206	0.379 vs. 0.212	0.026		0.228 vs. 0.162	0.199 vs. 0.156	**0.023**		W
	TMB	5.2 vs. 11.3	5.2 vs. 3.8	0.38		3.5 vs. 6.5	2.7 vs. 1.9	0.185		W

		Mean 2 vs. 3	Median 2 vs. 3	*P*	*n*	Mean 2 vs. 3	Median 2 vs. 3	*P*	*n*	
Mono-allelic vs. wildtype groups 2 vs. 3	Presence SBS36	0.02 vs. 0.00		0*.*35	44 group 2 vs. 2465 group 3	0.03 vs. 0.00		**<10**^**−7**^	221 group 2 vs. 12312 group 3	F
	Burden SBS36	273 vs. 17	0 vs. 0	0.062		68 vs. 2	0 vs. 0	<2 × 10^−16^		W
	Activity SBS36	0.011 vs. 0.001	0.000 vs. 0.000	0.062		0.006 vs. 0.000	0.000 vs. 0.000	**<2** **×** **10**^**−16**^		W
	Presence SBS18	0.50 vs. 0.27		**4.48** **×** **10**^**−4**^		0.13 vs. 0.04		**4.44** **×** **10**^**−7**^		*χ*^2^
	Burden SBS18	2751 vs. 1146	805 vs. 0	**9.59** **×** **10**^**−5**^		563 vs. 101	0 vs. 0	**8.88** **×** **10**^**−12**^		W
	Activity SBS18	0.122 vs. 0.054	0.072 vs. 0.000	**2.61** **×** **10**^**−5**^		0.025 vs. 0.007	0.000 vs. 0.000	**8.60** **×** **10**^**−12**^		W
	Burden C>A	6501 vs. 9542	4204 vs. 3668	**0.58**		7385 vs. 5736	1636 vs. 1422	0.016		W
	Activity C>A	0.214 vs. 0.173	0.215 vs. 0.172	**5.19** **×** **10**^**−5**^		0.165 vs. 0.153	0.144 vs. 0.133	**2.02** **×** **10**^**−5**^		W
	TMB	11.0 vs. 17.5	3.8 vs. 3.9	0.34		6.3 vs. 6.0	1.9 vs. 1.8	0.445		W

For signature presence/absence (binary), mean shows the proportion of positive individuals, whilst median has no utility and is not shown. P values are uncorrected and derived from univariable tests (F, Fisher’s exact; *χ*^2^; W, Wilcoxon). **Bold**, associations remaining significant in multivariable regression. More detailed data are in Supplementary Tables 1–7 and 13–16. Note that for CRC in particular, the means for some measures of mutation are higher in group 3 than group 2, whereas the medians are higher in group 2 than group 3, principally due to some MMRd tumours with very high TMB (Supplementary Tables 1 and 2).

Very few group 3 CRCs had acquired a somatic mutation specifically involving *MUTYH* and none had acquired bi-allelic somatic *MUTYH* mutations. It was common (frequency ~30%) for MSS CRCs to show heterozygous somatic allelic imbalance encompassing the *MUTYH* locus, but this generally reflected a 2:1 ratio of the parental copies of chromosome 1 in cancers with a near-triploid karyotype, rather than any targeting or true loss of *MUTYH*. Specifically, the 4.1% of MSS cancers with either a somatic deletion of 1p or a single, heterozygous somatic mutation had almost the same SBS18 prevalence as cancers with no change of any type at *MUYTH* (OR = 1.06, 95% CI 0.780–1.44, *P* = 0.765). All these CRCs were therefore analysed as part of the ‘*MUTYH* wildtype’ group 3.

### Associations between germline *MUTYH* genotype and somatic molecular features of CRCs from the 100kGP

**Fig. 4:**
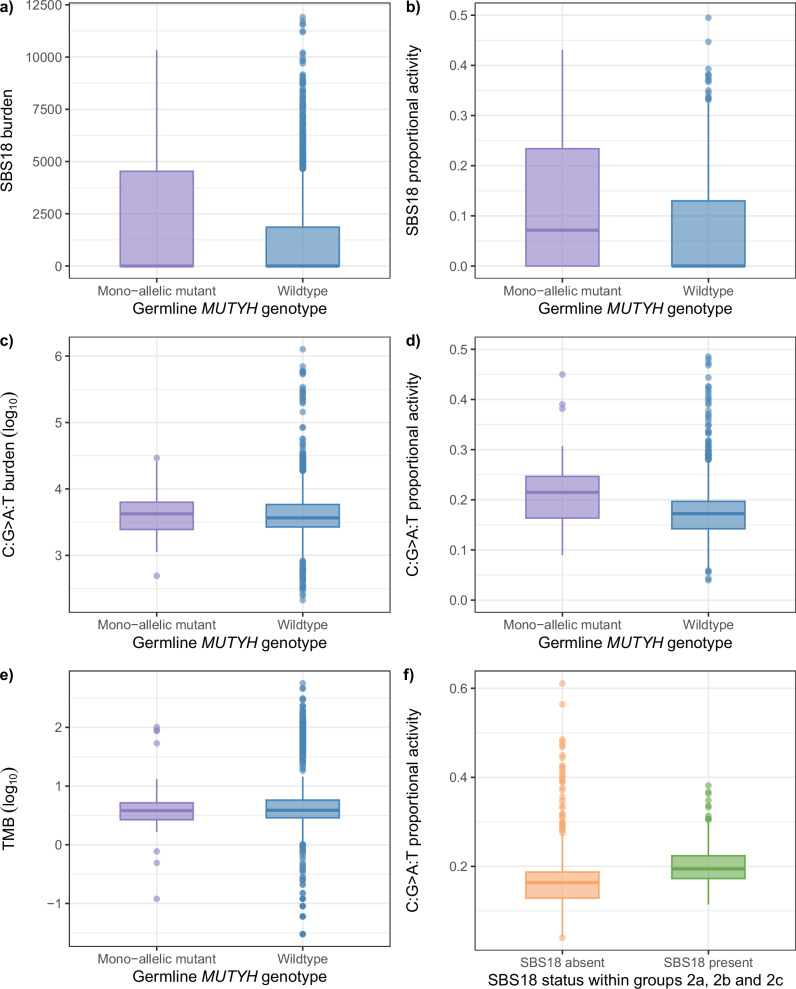
Associations in all 100kGP CRCs between measures of somatic mutation and germline heterozygote *MUTYH* status (group 2) versus vs wildtype (group 3). **a** SBS18 burden; **b** SBS18 activity; **c** C:G > A:T burden; **d** C:G > A:T activity; and **e** TMB (measured as somatic coding variants per Mb). **f** Shows the expected positive relationship between the presence of SBS18 and C:G > A:T activity within group 2; a weaker positive association was seen with C:G > A:T burden (not shown). Log scales are used to aid display in some cases. Nominally significant associations (*P* < 0.05) were present based on Wilcoxon tests for (**a**, **b**, **d**). Associations from Fisher’s exact, *χ*^2^, Wilcoxon and regression analyses are shown in Table 1 and Supplementary Tables 3–5.

Owing to the reported link between bi-allelic *MUTYH* mutations and SBS36, we searched initially for associations between germline *MUTYH* genotypes and mutational signatures SBS36 and SBS18 in the sporadic CRCs. We also tested for associations with measures of mutational load, specifically C:G > A:T substitution burden and activity, and total mutation burden (using somatic coding variants per megabase (SCVPM) as a measure). For clarity, we summarise the main results here, in Table 1 and in Figs. 3 and 4. Full supporting data are provided in Supplementary Tables 1–8.

First, compared with all other CRCs, bi-allelic germline *MUTYH* mutations (group 1) were positively associated with all three SBS36 metrics (presence, burden and activity), higher C:G > A:T burden and activity, and higher overall TMB (Fig. 3). There were no significant associations with SBS18. These results were largely confirmatory of previous studies^8,19^.

Second, in germline heterozygotes (group 2), there was no tendency for the wildtype copy of *MUTYH* to be lost over the mutant (OR = 0.530, 95% CI 0.026–8.30, *P* = 1.00, Fisher’s exact). The very small number of mono-allelic *MUTYH* carriers with somatic loss of the wildtype allele in their tumours (group 2a, *n* = 2) limited the comparison with groups 2b/2c, although C:G > A:T activity was a little higher in the former (Fig. 3). There was also evidence that the resulting MUTYH deficiency in the group 2a CRCs could specifically cause SBS36 rather than SBS18 (Supplementary Fig. 1). No mutations were assigned to SBS36 in mono-allelic *MUTYH* carriers' cancers that did not have loss of the wildtype allele (groups 2b/2c, *n* = 42).

Third, germline heterozygotes as a whole (group 2) had higher SBS18 metrics and C:G > A:T activity than *MUTYH* wildtype cases (group 3). Half of the 44 individuals in group 2 exhibited SBS18 in their CRC, representing a highly significant increase over group 3 (2.7-fold odds ratio, *P* = 4.48 × 10^−4^; Table 1, Fig. 4, Supplementary Tables 1 and 2). This association remained strong (2.7-fold odds ratio, *P* = 3.00 × 10^−3^) when the two group 2a CRCs were excluded, indicating that it was not driven by somatic loss of the wildtype *MUTYH* allele. SBS18 mean burden and activity were both about 2.5-fold higher in group 2 than in group 3 (Fig. 4). In comparison, SBS36 metrics were similarly low in groups 2 and 3. In multivariable analysis (details not shown), significantly higher SBS18 and C:G > A:T metrics were independently associated with group 2 (*versus* group 3), absence of mismatch repair deficiency (MMRd) and, like SBS18-positive *MUTYH-*wildtype CRCs^18^, cancer location in the proximal colorectum.

To avoid associations between mono-allelic *MUTYH* mutations and tumour features being confounded or obscured by the approximately six-fold increased SBS burden of MMRd cancers that is biased to C:G > T:A changes^20^, we repeated the above analyses in MSS/polymerase proofreading-proficient CRCs only (*n*=37 in group 2 and *n*=1875 ingroup 3). The associations between group 2 and increased SBS18 and C:G > A:T were generally very similar to those in the all-CRC analyses (Supplementary Tables 1–8). Of note, however, in MSS-only regression analyses corrected for the number of non-C:G > A:T mutations in each cancer, TMB was significantly increased in mono-allelic *MUTYH* carriers (group 2) compared with the *MUTYH*-wildtype individuals of group 3 (for SCVPM measure of TMB, *β* = 0.447, SE 0.223, *P* = 0.045; for total SBS burden, *β* = 1839, SE 725, *P* = 0.011). The equivalent association was not detected in an MMR-proficient-only analysis, perhaps unsurprisingly given the much higher background level of SBSs in those tumours (for SCVPM, *β* = −10.3, SE 5.99, *P* = 0.086; for total SBS burden, *β* = 1160, SE 1623, *P* = 0.475). Mono-allelic *MUTYH* carriers contributed 4.1% of all SBS18 mutations in MSS CRCs (Supplementary Table 2).

One of the reasons that we were careful to assess simpler metrics (e.g. C > A burden and activity) alongside SBS18 was to check that our conclusions were supported by the underlying base substitution data. Within group 2b (mono-allelic *MUTYH* mutations), SBS18-positive cancers overall had higher C:G > A:T activity than SBS18-negative tumours (*P* = 0.021, MSS-only multiple logistic regression analysis; Fig. 4f), although C > A burden, age and sex were not significantly different between the groups (*P* = 0.631, *P* = 0.410 and *P* = 0.598, respectively). We found that none of six MMRd cancers in this group was SBS18-positive, and we suspect that any SBS18 activity was obscured by the high overall SBS burden in MMRd tumours. In the remaining 16 SBS18-negative tumours, however, C:G > A:T mutation burdens and activities were very similar to those of group 3 CRCs (0.187 vs. 0.185 and 3580 vs. 3238, respectively, *P* > 0.2 for both). In the absence of evidence to the contrary, we therefore regarded the former as arising independently of *MUTYH*, perhaps because they developed in a low oxidative damage environment.

Given that MAP patients’ colorectal tumours are known to acquire extra C:G > A:T mutations in driver genes owing to bi-allelic MUTYH deficiency^13^, we tested whether the CRCs of mono-allelic mutation carriers (group 2) also tended to acquire C:G > A:T driver changes. This would be expected if the associated increased SBS18 activity occurred prior to, or early in, colorectal tumorigenesis. Based on a pre-specified analysis of the 20 most common CRC driver genes^18^, we found that group 2 patients’ CRCs did indeed carry an increased proportion of C:G > A:T driver changes (Table 2).

**Table 2:** Associations between driver mutation spectra in the 20 most frequently mutated CRC driver genes and mono-allelic *MUTYH* mutations

Driver gene mutation	*MUTYH* mono-allelic	*MUTYH* wildtype	Total	*MUTYH* bi-allelic
C:G > A:T	23	908	931	14
Other SBS	56	3776	3832	9
Total	79	4684	4763	23

Analysis was restricted to MSS cancers. OR = 1.708, 95% CI 1.046–2.79; *P* = 0.0306, *χ*^2^_1_ test. Data from the two MSS CRCs occurring in bi-allelic germline *MUTYH* mutation carriers are shown for comparison.

In an analysis focused solely on SBS18-positive CRCs, we investigated whether both the C:G > A:T mutations and other somatic molecular features of the cancers were similar in groups 2 and 3, so as to identify any distinctly different mechanisms of mutagenesis in the two groups. SBS18 burden/activity and C > A burden/activity were all significantly higher in group 2 in univariable analysis, broadly consistent with the all-CRC analysis (Supplementary Table 8), but the two groups were similar in terms of SCVPM, age, location, whole genome doubling and MMRd (Supplementary Tables 1–8). These results were confirmed in multivariable analyses (not shown). They were consistent with a simple model in which both groups are exposed to similar levels of oxidative DNA damage, but damage repair is less effective in group 2 patients.

### Associations between germline mono-allelic *MUTYH* mutations, SBS18 and C:G > A:T mutations are also present in CRCs from the Hartwig collection

We tested whether the associations between mono-allelic *MUTYH* mutations and mutational processes in the 100kGP CRCs were present in an independent data set. We derived germline *MUTYH* genotypes and somatic mutation data from 493 metastatic CRCs in the Hartwig study^21,22^. Although many of the tumours analysed had been subjected to genotoxic therapy, we still observed significantly higher SBS18 burden, SBS18 activity and C:G > A:T burden in the 14 CRCs from group 2 compared to the group 3 patients (Supplementary Table 9).

### Association study of CRC risk in carriers of germline *MUTYH* mutations and causality analysis

**Fig. 5:**
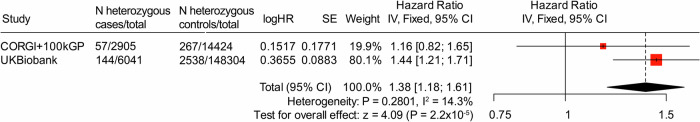
Inverse variance-weighted meta-analysis of association between CRC/polyposis and *MUTYH* heterozygote status (cases) in 100kGP and UKB. Black dots show point estimates of odds ratio (OR) for each study, with whiskers showing 95% CIs. Squares are proportional to study size. The red line shows the pooled OR and the diamond the pooled 95% CIs. See also supporting data from SNP microarray analysis Supplementary Table 10.

In order to explore the possibility that increased SBS18 activity and TMB led to a raised risk of CRC in germline *MUTYH* heterozygotes, we utilised two sets of CRC cases and controls with germline whole-genome sequencing (WGS): (i) UK 100,000 Genomes Project (100kGP, 10.6084/m9.figshare.4530893.v7) supplemented with our own CORGI study; and (ii) UK Biobank (UKB)^23^ (Fig. 5). We filtered participants to ensure ancestral matching and exclusion of multiple close relatives, and removed individuals with probable bi-allelic germline *MUTYH* mutations. Common *MUTYH* polymorphisms (allele frequency >0.025) and other variants not predicted to be pathogenic from ClinVar were also excluded. Meta-analysis of the cohorts, comprising 8,946 cases and 162,728 controls, demonstrated an association between *MUTYH* heterozygote status and CRC (OR = 1.38, 95% CI 1.18–1.61; *P* = 2.2 × 10^−5^; Fig. 5 and Supplementary Table 10).

Since about half the CRCs from germline *MUTYH* heterozygotes did not have SBS18, we examined candidate modifier SNPs for negative effects on *MUTYH* expression that might help to ‘reveal’ haploinsufficiency in heterozygotes’ CRCs^24^. The SNP set was restricted to a ~2 Mb window flanking *MUTYH* (hg38 chr1:44,329,242-46,339,970) and to common SNPs from dbSNP155 (https://genome-euro.ucsc.edu/cgi-bin/hgTrackUi?hgsid=407686972_tMiyAfTray8NrAweTgTfYMqHxAx6&db=hg38&c=chr1&g=dbSnp155Composite), and included several strong expression- and splice-quantitative trait loci for *MUTYH* (https://www.gtexportal.org/home/gene/MUTYH). However, we found no evidence of SNP alleles associated with the presence of SBS18 in mono-allelic *MUTYH* carriers (uncorrected *P* > 0.05 in all cases, details not shown). Whilst we cannot exclude genetic modifiers elsewhere in the genome, functional *MUTYH* haploinsufficiency, perhaps coupled with environmental exposure to oxidative damage, thus seems the most likely causal mechanism.

The principles of Mendelian randomisation^25,26^ indicated that mono-allelic germline *MUTYH* mutations are probably causal for increased SBS18, and hence that SBS18 may be causal for CRC. A causal link between SBS18 and raised CRC risk was supported by a Wald ratio estimation for the SBS18-CRC association (*mrratio* command in STATA, β_SBS18-CRC_ = 0.177, SE 0.085, OR = 1.19, *P* = 0.037)^27^. We also performed a comprehensive check for sources of collider bias and other confounders that could have led to a spurious association between mono-allelic *MUTYH* mutations and SBS18 in CRC, including factors such as genetic ancestry and geographical location that could, in principle, co-vary with germline genotype, but no evidence of any such confounder was found (Supplementary Tables 11 and 12).

### Mutational features in germline *MUTYH* heterozygotes in the 100kGP all-cancer cohort

The findings from CRC suggested that the effects of *MUTYH* heterozygosity might be pervasive, in that germline heterozygous genotypes could lead C:G > A:T mutations to accumulate in many normal and/or tumour cell types. We therefore examined SBS18, SBS36 and C:G > A:T mutations in the 100kGP All-cancer data set, which comprised 15,223 patients and tumours from multiple tissue locations. Owing to the inherent variation among these patients, we took a conservative approach and restricted this part of the study to assessing the three major findings from CRC (that is, higher SBS36 in group 1 than 2 and 3, occasional SBS36 in group 2a compared with 2b/c, and higher SBS18 in group 2 than 3). We here present data from analyses of all cancer types and of all cancers except CRC (‘CRC-excluded’) (Table 1, Fig. 3, Supplementary Tables 13–16 and Supplementary Figs. 2 and 3).

We first noted that CRCs generally had much higher SBS18 (and somewhat higher SBS36) metrics than other cancer types (Fig. 3, Supplementary Tables 1, 2 and 13–16), including approximately three-fold higher SBS18 prevalence, but 10-fold higher SBS18 activity and C:G > A : T burden. In the germline *MUTYH* heterozygotes (group 2), we identified 25 non-CRC cancers with LOH of the wildtype or mutant *MUTYH* allele (groups 2a and 2b, respectively). As in CRC, there was no tendency for the wildtype copy of *MUTYH* to be lost over the mutant (OR = 0.778, 95% CI 0.220–2.786, *P* = 0.778, Fisher’s exact). Furthermore, the 10 cancers in which the wildtype allele was lost (group 2a) were from eight different tissue or cell types, providing no evidence that loss of the wildtype conferred a selective advantage in specific tumour types. No cancer had bi-allelic somatic *MUTYH* mutations (including LOH and homozygous deletions).

Overall, despite the multiplicity of tumour types in the CRC-excluded analysis and the less frequent C:G > A:T changes, the CRC-excluded data strongly supported the findings from CRC on C:G > A:T mutations and mutational signatures (Table 1 and Supplementary Tables 14–17). An excess of SBS36 and SBS18 was present in bi-allelic *MUTYH* mutation carriers’ tumours (group 1 vs. 2&3). SBS36 was increased in the tumours of heterozygotes that acquired somatic loss of the germline wildtype allele (group 2a vs. 2b/c), with accompanying increased C:G > A:T activity. There was particular support for the association between heterozygous germline *MUTYH* mutations and SBS18 (group 2 vs. 3), with effect sizes (as measured by odds ratios) similar to the CRC-only analyses (Table 1). In fact, mono-allelic *MUTYH* carriers contributed 8.2% of all SBS18 mutations in CRC-excluded cancers, a significantly higher proportion than in MSS CRCs (*P* < 10^−7^, Supplementary Tables 1, 2, 15 and 16).

We performed specific analyses of cancers of the upper gastrointestinal tract (sometimes found in MAP), breast (common female-predominant sporadic cancer), prostate (common male-predominant sporadic cancer) and endometrium (major part of Lynch syndrome and polymerase proofreading polyposis). The analyses were limited by smaller sample sizes and/or lower SBS18 metrics than CRC and All-cancer, but provided good support for higher SBS18 (or SBS36) and/or raised C:G > A:T levels in (i) group 2a vs. 2b/c and (ii) group 2 vs. 3 (Supplementary Tables 16, 18 and 19).

In summary, despite the many different tumour types in the All-cancer and CRC-excluded analyses, the positive effects of bi-allelic and mono-allelic germline *MUTYH* mutations on mutational processes (SBS36, SBS18 and C:G > A:T changes) were clearly present, as they were in CRC. Given the much higher baseline SBS18 and C:G > A:T metrics in CRCs than other cancers, the importance of germline heterozygous *MUTYH* mutations, and the resulting C:G > A:T changes and SBS18 may be much greater as a driving force of tumorigenesis in CRC. Nevertheless, the SBS18, SBS36 and C:G > A:T data suggested that some increased risk of other cancers in bi-allelic and heterozygous *MUTYH* mutation carriers might exist.

### The risks of multiple cancer types in carriers of heterozygous germline *MUTYH* mutations

One of the purposes of the current study was to determine whether the associations between *MUTYH* genotype and cancer risk reported by others were also present in the large UK cancer data sets. After including only unrelated individuals of European ancestry and excluding CRC or polyp cases, we identified 13,252 cases with any cancer and 14,407 cancer-free controls in the 100kGP, plus 14,813 cases and 9599 controls from UKB exome sequencing. Bi-allelic *MUTYH* mutations were associated with no cancers other than CRC (Supplementary Tables 20–25).

For assessing associations between All-cancer or CRC-excluded risk and *MUTYH* germline heterozygosity, there was estimated 70% power to detect an association at nominal *P* = 0.05 if *MUTYH* heterozygosity conferred a relative risk of 1.20 (https://csg.sph.umich.edu/abecasis/gas_power_calculator/index.html). However, there was correspondingly no increased risk of CRC-excluded cancers in a meta-analysis of 100kGP and UKB data with age and sex as co-variables (OR = 0.93, 95% CI 0.81–1.07, *P*_*meta*_ = 0.31; *P*_het_ = 0.74, *I*^2^ = 0%), or indeed with All-cancer risk (OR = 0.97, 95% CI 0.85–1.10, *P*_*meta*_ = 0.61; *P*_het_ = 0.72, *I*^2^ = 0% (Supplementary Tables 20–25). Since there have been variable reports that *MUTYH* heterozygosity confers risks of cancers of specific organs other than CRC^7,14,16,28–35^, we also assessed these in 100kGP and UKB. No cancer type achieved *P* < 0.10_*meta*_ (Supplementary Tables 24 and 25).

### De novo mutations in the offspring of *MUTYH* heterozygotes

We searched for effects of *MUTYH* heterozygosity on germline de novo mutation (DNM) rates in parent-offspring trios from 100kGP^36^. We found 206 trios in which one parent was a *MUTYH* heterozygote. Compared with 11,308 *MUTYH* wildtype control trios, there was a small, but significantly increased total de novo mutation burden in the offspring of *MUTYH* carrier parents assessed using *standard criteria* (median 948 vs. 915, *P* = 0.016), but not using *stringent criteria* (median 69 vs. 70, *P* = 0.55), which is the measure recommended by 100kGP (see ‘Methods’ for link to definitions). We also specifically assessed de novo C:G > A:T mutations and found no association (median 6 for both groups, *P* = 0.783, stringent criteria). We concluded that any effect of mono-allelic *MUTYH* mutations on DNMs was likely to be small at most (Supplementary Table 24).

### Other base excision repair heterozygotes may be predisposed to specific mutational signatures

We explored whether the association between germline heterozygotes, somatic mutations and cancer risk was a special feature of *MUTYH*, or was present for other BER genes. *MBD4* was chosen for study, because its recessive syndrome is genetically similar to MAP and includes multiple polyps and CRC, and no heterozygote risk of colorectal tumours has been established^37–40^. Bi-allelic germline *MBD4* mutations predispose to increased CpG>TpG changes, resulting in an SBS1-like signature. Apart from a raised (yet very small absolute) risk of uveal melanoma in *MBD4* heterozygotes owing to somatic loss of the germline wildtype allele^41^, no increased cancer risk in *MBD4* heterozygotes is known.

We found the frequency of LoF germline mutations in *MBD4* to be ~0.1%, over an order of magnitude lower than that for *MUTYH*. In 100kGP All-cancer, after excluding two cancers with second hits, 14 *MBD4* germline mutation carriers were present. Compared with over 14,000 All-cancer *MBD4-*wildtype cases, *MBD4* germline heterozygosity was associated with higher SBS1 burden, SBS1 activity and C:G > T:A activity in tumours at nominal *P* < 0.05 in univariable analysis. SBS1 burden (*P* = 0.0143), SBS1 activity (*P* = 0.0096) and C:G > T:A burden (*P* = 0.00201) remained significantly associated in multivariable analysis (Supplementary Table 27a). In CRC-excluded cases, mono-allelic *MBD4* status was associated in univariable analysis with SBS1 burden and activity, whereas associations in the smaller CRC data set did not reach statistical significance. Given the evidence that *MBD4* heterozygotes can cause an excess of C:G > T:A mutations, we revisited the question of whether such individuals are predisposed to CRC in 100kGP+CORGI and UKB. Whilst further studies are required, we found an increased risk of CRC in the mono-allelic *MBD4* mutation carriers (OR = 1.75, *P* = 0.020; Supplementary Table 27b).

## Discussion

Our study is one of the biggest yet to consider cancer risk in carriers of germline mono-allelic *MUTYH* mutations. Our results suggest that these individuals do have a moderately (~1.4-fold) increased risk of CRC. We took steps to exclude bias in the analysis, to the greatest degree possible. We specifically believe it unlikely that cryptic second germline *MUTYH* mutations in the mono-allelic mutation carriers (group 2) can explain the association we find between mono-allelic *MUTYH* mutations and SBS18 – this is partly because we used WGS data and partly because SBS36 was highly specific to cancers with bi-allelic germline *MUTYH* mutations or somatic loss of the wildtype allele (group 2a). No group 2b/c CRCs (without somatic loss of the wildtype allele), and only 3/253 group 2 non-CRCs, showed SBS36, despite its similarity to SBS18 and thus hypothetical possibility of chance mis-assignment (Supplementary Tables 1, 2, 14 and 15). Our association study did not support previously reported increased risks of extra-colonic cancers in carriers of mono-allelic or bi-allelic germline *MUTYH* variants, although very small effects remain possible^16,17,29,33,34,42–45^.

Other groups have proposed that somatic loss of the wildtype *MUTYH* allele causes increased cancer risk in germline *MUTYH* heterozygotes. Our findings differ considerably from those studies, which employed a variety of sample types, molecular platforms and algorithms to assess LOH. For example, Paller et al.^17^ studied FFPE samples from >350,000 Foundation Medicine tumours, analysed on a 311- or 324-gene diagnostic panel. Constitutional DNA was absent, and hence *MUTYH* variants were assigned as germline or somatic using a bespoke algorithm^46^, and SBS18 was quantified using a specially-designed, specific score. In contrast to our data, Paller et al. reported loss of the germline wildtype allele in 12% of mono-allelic mutation carriers (our group 2a), but no tumours had loss of the mutant allele (our group 2b). It is likely that the differences between the Paller et al work. and our study lie in a combination of sample types, molecular methods and available data analysis platforms. In our opinion, the 100kGP data show the benefits of tumour genomics in determining mechanisms of increased CRC risk, by allowing the full assessment of mutational spectra and signatures that can act as causal mediators.

Our data do show that somatic LOH of the wildtype allele can occur in the tumours of a small minority of mono-allelic *MUTYH* carriers (group 2a), and that this sometimes elicits the MAP signature SBS36. However, whilst somatic loss of the wildtype *MUTYH* allele in ~5% of mono-allelic mutation carriers could in theory explain the estimated increased risk of CRC in group 2 as a whole, the ‘second hit’ mutation should be selected and thus much more common than loss of the mutant allele (i.e. group 2a should be much larger than group 2b). We found no evidence of this. Furthermore, about 90% of mono-allelic carriers showed no somatic loss in their CRCs (group 2c), yet still exhibited the associations with SBS18. Our evidence thus indicates that haploinsufficiency, acting through raised SBS18 and hence increased TMB and driver gene mutations, is the main mechanism of increased CRC risk in all mono-allelic *MUTYH* carriers. A plausible scenario is that exposure to extrinsic or intrinsic oxidative stress periodically overwhelms the capacity of a single functional *MUTYH* allele to repair the resulting DNA damage. In our opinion, much of the SBS18-associated DNA damage in tumours from carriers of mono-allelic germline *MUTYH* mutations (group 2) may occur in precursor lesions or even normal cells prior to being ‘revealed’ in the clonally expanded cancer cells. This can explain the similar SBS18 levels in cancers with and without second hits at *MUTYH*, and the lack of excess SBS18 in CRCs with somatic mono-allelic deletion of one copy of *MUTYH* and no germline mutation. The evidence that somatic loss of *MUTYH* is not advantageous for tumorigenesis appears to conflict with the evidence that moderate hypermutation in *MUTYH* germline heterozygotes increases tumour risk. However, this may simply reflect the near-ubiquitous effects of germline alleles that are selected at the level of the organism, whereas somatic mutations in ‘caretaker genes’ have to survive strong competition from thousands or millions of other cells in the same tumour, even at the pre-malignant stage^47,48^.

The detection of increased SBS18 and C:G > A:T somatic mutations in the All-cancer and CRC-excluded tumour sets was both important and not fully expected. Of note, however, Moore et al.^49^ found SBS18 to be active in 19/29 normal cell types, consistent with our findings. The lower baseline SBS18 levels in the CRC-excluded tumours may limit the effects on cancer risk, consistent with our failure to detect raised risk of extra-colonic malignancies in our data^7,16,30,32^. We also found no evidence of increased de novo mutations in *MUTYH* heterozygotes, again consistent with Moore et al.^49^, who did not detect SBS18 in seminiferous tubules.

In conclusion, *MUTYH* heterozygotes have an increased risk of CRC owing to haploinsufficiency, increased C:G > A:T mutations and raised activity of signature SBS18. We have shown more generally that non-rare germline variation in DNA repair genes can lead to specific mutational signatures and hypermutation in multiple tumour types, without somatic second hits being required. The magnitude of increased CRC risk is moderate in *MUTYH* heterozygotes—although higher than most common risk polymorphisms—and it is possible that some such individuals exposed to high levels of reactive oxygen species^50^ are at more elevated risk. It is plausible that *MBD4* heterozygotes also have an increased risk of CRC through similar mechanisms, although the rarity of these individuals mandates some caution in this regard. In total, we estimate that about 5% of the population could be germline heterozygotes for apparently recessive DNA repair genes^51^ (https://gnomad.broadinstitute.org/). Further large studies will be required to confirm this estimate and explore the clinical utility of identifying such individuals. Whilst the *MUTYH* (or *MBD4*) alleles appear to constitute a valid instrumental variable for CRC risk, acting through the intermediate phenotype of SBS18 (or SBS1), it seems that the net effect on risk is absent or undetectable for many cancer types. It is arguably reassuring that a two- to three-fold increase in the activity of a common signature, like SBS18 in CRC, has a 1.4-fold effect on cancer risk, and that effects on the risk of other cancers are undetectable. Nonetheless, we contend that polygenic risk scores for CRC should include *MUTYH* genotypes. Furthermore, despite past controversies^52^, measures to reduce oxidative damage exposure could indeed be an effective CRC prevention strategy in general, as long as suitable agents can be developed.

## Methods

### 100kGP and CORGI: CRC and multiple polyp cases and controls

Cases were individuals with colorectal carcinoma and/or multiple colorectal polyps who were recruited to (i) the 100kGP colorectal cancer domain owing to resection or biopsy of a colorectal carcinoma, (ii) the 100kGP familial colorectal cancer or multiple polyp domain, or (iii) the CORGI study (familial, early-onset or multiple CRCs and/or polyps). An individual maximum pairwise kinship coefficient threshold of 0.01625 was applied and individuals had predicted European ancestry >0.99. For each case, WGS data from constitutional DNA to median depth >30X were obtained using the Illumina Hi-Seq platform, together (in category (i)) with paired frozen cancer sample WGS to median depth >100X in many cases. Data from relatives of patients included in release version 14 of the rare disease domain in the 100kGP were used to create the control cohort of individuals. Included individuals had no clinical record of cancer (of any site), or a condition closely linked to cancer or malignancy (including not only colorectal polyp(s), but also other benign tumours or clonal haematopoiesis). Ancestry and kinship filters were applied as for 100kGP cases.

The CORGI and CORGI 2 studies were approved by Southampton and SW Hampshire and South Central Research Ethics Committees in the UK with references 06/Q1702/99 and 17/SC/0079. Local ethical review board approval was provided by the following NHS Foundation Trusts: Great Ormond Street Hospital; Newcastle upon Tyne Hospitals; Guy’s & St Thomas’s; North Cumbria University Hospitals; Cambridge University Hospitals; Leeds Teaching Hospitals; Royal Free Hampstead; South Tees Hospitals; Oxford University Hospitals; West Middlesex University Hospital; Nottingham University Hospitals; Eling Hospital; Rotherham; North West London Hospitals; Royal Liverpool and Broadgreen; University Hospitals of Leicester; Birmingham Women’s and Children’s; University Hospitals Birmingham; Manchester University Hospitals; Southampton University Hospitals; Royal Devon University Healthcare; and University Hospitals Bristol. Ethical approval for collection and analysis of 100kGP patient samples was obtained from the HRA Committee East of England–Cambridge South Research Ethics Committee (REC reference 14/EE/1112). All participants provided written, informed consent. All research was performed in accordance with the Declaration of Helsinki.

### UKB cases and controls

WES data were obtained under UK Biobank project ID 19655 and WGS data were obtained under project ID 155012. All individuals with a current diagnosis or personal history of colorectal cancer were identified based on International Classification of Diseases ICD-10 codes C180, C182-189, C19 or C20 using data field 40,006 or ICD-9 codes 1530–1534 or 1536–1541 using data field 40,013. We further included only individuals with tumour behaviour described as ‘malignant, primary site’ in data field 40,012 and with histology information available in data field 40,011 to confirm the CRC diagnosis. Cancers which were self-reported only (provided in data field 20001) could not be validated and were therefore excluded. Patients with colorectal polyps only were excluded, due to a lack of details on the polyp number and size. We then applied genetic ancestry and kinship filters to select for Europeans and unrelated individuals using the same threshold values as the other cohorts (>0.99 and <0.01625, respectively). For UKB controls, we excluded any individuals with an ICD-9 or ICD-10 cancer, or self-reported cancer diagnosis (provided in data fields 40,013, 40,006 and 20,001, respectively), or reports of colorectal polyp(s) based on ICD-10 codes D12, D120, D121, D122, D123, D124, D125, D126, D127, D128 or D129) in data fields 41202 and 41204, or ICD9 codes 211, 2111, 2112, 2113, 2114, 2115, 2116, 2117, 2118 or 2119 in data fields 41,203 and 41,205, or code 1460 in data field 20002. We also removed all individuals with reported history of cancer in either parent or any sibling of the cancer types with data available (breast, lung, bowel or prostate cancers). We then applied genetic ancestry and kinship filters as above. UK Biobank was approved by North West - Haydock Research Ethics Committee 21/NW/0157, IRAS project ID: 299116. All participants provided written informed consent. All research was performed in accordance with the Declaration of Helsinki.

### 100kGP and UK All-cancer case cohorts

From all unrelated individuals from the 100kGP All-cancer domain with European genetic ancestry, we selected participants with a diagnosis of a malignant neoplasm described by ICD-O-3 in their clinical history. This All-cancer cohort used individuals only from the 100kGP (no additional WGS data was included) and did not contain participants with only colorectal polyposis. From UKB, we selected participants with a malignant neoplasm using ICD9 and ICD-10 data fields 40,013 and 40,006, respectively, and we then converted these into ICD-O-3 codes. After genetic ancestry and kinship filters, 12,694 UKB cases remained. In both 100kGP and UKBioBank, we grouped cancers into 20 organ sites using the SEER recode paradigm (https://seer.cancer.gov/siterecode/icdo3_dwhoheme/index.html), whilst keeping colorectal cancers, upper gastrointestinal tumours, and cancers of the liver and intrahepatic bile duct as three separate categories for our analyses. We also selected for more specific tumour subtypes of interest. In 100kGP, this was performed by matching for the appropriate specific terms for tumour site and organ of origin in histological and other clinical records. In UKB, we extracted patient information with the following ICD codes, in order to facilitate comparisons with previous studies:

- Adrenocortical carcinoma (749 in ICD9; C740 in ICD10)
- Oesophageal carcinoma (1505 or 1509 in ICD9; C150-C159 in ICD10)
- Prostate adenocarcinoma (1850 in ICD9; C61 in ICD10)
- Renal clear cell (1890 in ICD9; C64 in ICD10)
- Sarcoma (1710-1719 in ICD9; C490-C499 in ICD10)
- Acute myeloid leukaemia (AML) (2050 in ICD9; C920 in ICD10)
- Uveal melanoma (1900, 1905, 1906 or 1909 in ICD9; C693 or C694 in ICD10)

All research was performed in accordance with the Declaration of Helsinki.

### Identifying deleterious germline variants in *MUTYH*

For 100kGP data, germline variants called and filtered by Starling (v2.4.7) within the Illumina WGS workflow were already available in the 100kGP research environment using the GRCh38 reference genome^53^. For UKB, germline variant calls and filtering information from DeepVariant (v0.10.0) were already available^54^. For both GEL and 100kGP, we selected all variants that passed the variant calling filtering criteria and intersected with the chromosomal location of *MUTYH*. We then re-annotated all variants using Ensembl VEP (v109.3)^55^ to obtain the full predicted functional consequences. We filtered in protein truncating variants (PTVs), and then added missense and any other variants annotated as pathogenic or likely pathogenic in ClinVar (https://www.ncbi.nlm.nih.gov/clinvar/?term=MUTYH)^56^. Whilst a small number of variants had conflicting interpretations of pathogenicity in ClinVar, closer inspection showed that the great majority of these were most likely to be pathogenic. Variants predicted by SpliceAI to have a splice donor or acceptor functional probability score > 0.8 were annotated as pathogenic, as the recommended threshold^57^. Germline structural variants were search for using MANTA. Individuals homozygous for a predicted (likely) pathogenic variant were classified as having bi-allelic *MUTYH* mutations, as were those with two different predicted (likely) pathogenic variants. Individuals with a single pathogenic germline variant of heterozygous genotype were classified as having mono-allelic loss of gene function, and the remainder of the cohort were categorised as germline wildtype for *MUTYH*.

### Somatic mutation analyses in 100kGP tumours

To investigate how germline mutations in *MUTYH* influence tumorigenesis in colorectal cancer and other cancer types, we identified patients with somatic sequencing data in 100kGP. This was not restricted to unrelated participants or those with European genetic ancestry. For ‘second hits’ and other somatic mutations at *MUTYH*, including somatic copy number changes and copy-neutral LOH (cnLOH), we used Battenberg (v2.2.8)^58^, where these data were available, or ASCAT (v3.0.0)^59^. Read depths of the reference and alternative (*MUTYH* mutant) alleles were also assessed in tumour (after correcting for purity) and normal sequence, particularly for determining whether the mutant or wildtype copy of the *MUTYH* gene was lost in tumours with LOH. LOH was scored when one copy of the *MUTYH* locus was absent in the cancer, with cnLOH (zero copies of one allele) if the other chromosome was also at the modal number in that tumour. We detected no homozygous deletions or high-level amplifications, and thus all other copy number states were scored as ‘no loss’ (NL). We also searched for pathogenic somatic mutations in *MUTYH* using existing somatic variant results from Strelka (v2.4.7).

### Identifying additional DNA repair deficiencies in cancer genomes

Microsatellite instability (MSI) was assessed using mSINGS^60^ as a proxy for MMRd. The multiplier parameter (the number of standard deviations away from the baseline required to call instability) was configured to 2.0. The maximum fraction of unstable sites permitted in order to call a cancer MSI-negative was set to 0.2, and the minimum proportion of unstable sites to call a sample MSI+ was also 0.2. The selection of the sites used and the success of the algorithm in distinguishing MSI+ and MSI-negative CRCs in the 100kGP data set has been described previously^18^. We also identified all tumours with known pathogenic germline or somatic mutations in the DNA polymerase genes *POLE* or *POLD1*^61^; these were classified as polymerase-proofreading (POL)-deficient.

### Mutational spectra and signature analysis

Tumour mutation burden (TMB) for each of the tumour samples was defined as the total number of pass-filtered non-synonymous SNVs and indels shorter than 50 base pairs, divided by the total length of the coding sequence (32.61 Mb) (see https://re-docs.genomicsengland.co.uk/cancer_clinical/). We extracted all single base substitution (SBS) mutations present in each tumour in their trinucleotide (96-channel) context using the tool SigProfilerMatrixGenerator (v1.2.1)^62^ and then inputted the data to SigProfilerAssigment (v1.2.1)^63^ in order to assign corresponding mutational signatures from the COSMIC (v3.3) reference database. SigProfiler was implemented within Python v3.6.8 using GRCh38 as the reference genome and all other parameters at the recommended default values. Mutations in each channel or signature were generally assessed in three ways: (a) *prevalence* (presence at any level >0% or absence) in a cancer; (b) *burden* (absolute number of a mutation type in a cancer); and (c) *proportional activity* (the total burden of a defined class of mutation relative to all mutations of that type). Examples of measures of proportional activity (sometimes referred to simply as ‘activity') included C:G > A:T mutations relative to all SBSs or mutations assigned to signature SBS18 relative to all mutations assigned to a SBS signature. The three mutational measures co-vary, but each has specific strengths. Prevalence is non-quantitative, but is useful for determining whether a specific signature (and underlying mutational process) is active within a tumour; it appears to be a relatively sensitive and specific measure (e.g. despite its resemblance to SBS18, SBS36 is only very rarely found outside the context of MUTYH deficiency). Burdens are particularly useful for identifying hypermutant tumours, owing to genomic instability, environmental exposure or unknown factors. Proportional activity is useful when correcting for background burdens to identify the most active processes in a single tumour; one use is to identify important mutational processes that are not found by crude TMB measures owing to ‘competing’ mutational processes (*e.g*. in a process of parallel evolution, different CRCs may derive the mutations they require from different sources without major effects on TMB).

### Colorectal cancer driver gene analysis

To examine the effects of somatic *MUTYH* genotypes on colorectal tumorigenesis, we extracted driver mutations from the following 20, relatively common established CRC driver genes in 100kGP version 19 (*ARID1A, BCL9, ZFP36L2, TGFBR2, PIK3CA, FBXW7, APC, BRAF, KMT2C, PTEN, TCF7L2, ATM, BCL9L, KRAS, TP53, RNF43, SOX9, SMAD4* and *ASXL1*)^18^. Specifically, we extracted small mutations (almost all SBS or indel) and assigned pathogenicity, including bi-allelic mutations for tumour suppressor genes.

### De novo mutations

WGS data^36^ were available from 13,912 trios (both parents and a child) within the 100kGP project, accompanied by sets of de novo mutations in the child that were present in neither parent and were presumed to have arisen in the parental gametes, or perhaps at an early stage of the child’s development. We assessed de novo mutation burdens in these trios according to the parental *MUTYH* variant status using data mapped to genome build 38. Further details of the 100kGP de novo mutations project and their recommendations for use of the *stringent m*utation sets can be found at https://re-docs.genomicsengland.co.uk/de_novo_data/.

### Germline *MBD4* mono-allelic mutation carriers

Analysis was generally performed as for *MUTYH*. We excluded MMRd tumours to avoid background somatic mutations that are known to occur, sometimes as sub-clonal events, within a short coding repeat in *MBD4*. One mono-allelic mutation carrier with a second hit by LOH in their tumour was also excluded from the analysis of associations with somatic mutational features. Seventeen uveal melanoma cases were identified and excluded, although none were heterozygous for *MBD4*. No bi-allelic *MBD4* mutation carriers were found. SBS1 burden, SBS1 activity, C > T burden, C > T activity and SCVPM were used as outcomes to assess the effects of germline mono-allelic *MBD4* mutations in All-cancer analyses only, owing to limited power in CRC-only analyses.

### Statistical analysis

All statistical analyses were performed in R (v4.2.1). All figures were produced using the R package ggplot2. Germline analyses used multiple logistic regression for the binary case-control outcome, incorporating *MUTYH* status as the chief explanatory variable, and also age and sex. Numbers of individuals used in each analysis are reported in the Figures, Tables and Supplementary Tables. Meta-analyses were performed using fixed effects, inverse variance weighted methods in the package metafor (v4.6), based on 2-tailed tests. For somatic mutation data, we reported data from up to three measures (presence, burden and activity), as described in the Results. Presence was omitted when the measurement was trivial (e.g. all cancers had at least one C:G > A:T mutation). Categorical analyses (mostly 2 × *n* tables) were generally performed using two-tailed χ^2^ tests or, when a value in any cell was <10, by Fisher’s exact tests, unless stated to the contrary. For assessing binary explanatory variables and quantitative outcomes, Wilcoxon tests and linear regression were generally used. Linear regression was used for most other multivariable analyses. Since many variables were non-normally distributed, models with robust standard errors (using the *lm_robust* command in the *estimatr* package in R) were used for non-normal distributed, proportional or skewed data. Most notably, activities of mutational signatures (although not individual channels such as C:G > T:A) were bimodal, with one maximum at zero and another within an approximately normal distribution at (>0, <1), this being a result of the methods used to derive signatures. ln(odds ratios) (β), standard errors and *P* values were reported. Covariables in these analyses included combinations of the following: participant age, sex, proximal or distal tumour location (for CRC analyses), hypermutation (from MSI or polymerase mutations), prior cancer genotoxic treatment, TMB, tumour purity, and sequencing from FFPE specimen or non-PCR-free library. We used the R package broom (v1.0.6) to select the combination of variables with the lowest output Akaike information criterion (AIC) value, which best explains the data while limiting overfitting, and combined this with reverse stepwise analysis to ensure that all variables remaining in the final model had nominal *P* < 0.05. In most somatic molecular analyses—such as those involving signature presence, burden and activity—co-variation among both explanatory and outcome variables was both common and complex, and the use of FDR may thus lead to over-correction. We decided, therefore, to report unadjusted *P* values from 2-tailed tests. As a result, we endeavoured to interpret association statistics cautiously.

## Supplementary information

Supplementary Information

## Data Availability

Genomics England permits access to 100kGP data used for this study subject to the following conditions. Research on the de-identified patient data used in this publication can be carried out in the Genomics England Research Environment subject to a collaborative agreement that adheres to patient led governance. All interested readers will be able to access the data in the same manner that the authors accessed the data. For more information about accessing the data, readers may contact research-network@genomicsengland.co.uk or access the relevant information on the Genomics England website, https://www.genomicsengland.co.uk/research. This research has been conducted using the UK Biobank Resource under application numbers 19655 and 155012. UK Biobank data are available through the UK Biobank (http://www.ukbiobank.ac.uk/) upon application, with permission of UKB’s Research Ethics Committee. Other data will be made available by the lead researchers to collaborating researchers, subject to ethical permissions and formal agreement.
