# Supplementary material for: Germline haploinsufficiency of MUTYH causes mutational signature SBS18 in multiple tumour types and specifically raises colorectal cancer risk
Source: NPJ Precis Oncol. 2026 May 6;10:275. doi: 10.1038/s41698-026-01425-x (PMC13358035; doi:10.1038/s41698-026-01425-x)
Supplement: Supplementary file 1 — Supplementary Information [file 41698_2026_1425_MOESM1_ESM.pdf]

Supplementary Information

Supplementary Tables	
1	Summary of somatic molecular features of 100kGP CRCs
2	Summary of somatic molecular features of 100kGP MSS CRCs
3	SBS36 & SBS18 presence and absence in germline *MUTYH* mutation groups in cases with CRC, Pan-Cancer & CRC-excluded from the 100kGP
4	Pairwise associations in univariable analyses between germline *MUTYH* genotypes & cancer molecular features in 100kGP CRCs, All-cancer & CRC-excluded
5	Multiple regression analysis for associations between germline *MUTYH* genotypes & mutational processes in 100kGP CRCs
6	Associations between CRC location (proximal v distal colorectum), MSI and germline *MUTYH* genotype (group 2 vs group 3)
7	Associations between germline *MUTYH* genotypes & molecular features in 100kGP MSS CRC-only analyses
8	Comparison between SBS18-positive CRCs from mono-allelic *MUTYH* mutation carriers and *MUTYH*-wildtype individuals
9	Associations between mono-allelic germline *MUTYH* mutations & molecular features in 493 metastatic CRCs from Hartwig study
10	Exome SNP array data for *MUTYH* p.Gly382Asp and Tyr179Cys
11	Exploration of potential confounders that could have caused the association between mono-allelic *MUTYH* mutations and SBS18 in 100kGP CRCs
12	Geographical origins of the patients from different regions of England
13	Non-CRC cancers analysed for associations between *MUTYH* genotypes and somatic molecular features
14	Summary of somatic molecular features of 100kGP All-cancer tumours
15	Summary of somatic molecular features of 100kGP CRC-excluded tumours.
16	Summary of somatic molecular features of Upper GI cancers.
17	Multiple regression analysis of 100kGP All-cancer and CRC-excluded data sets
18	Pairwise associations in univariable analyses between germline *MUTYH* genotypes and molecular features in breast, prostate and endometrial cancers
19	Multiple regression analysis of Upper GI cancers.
20	Associations between germline *MUTYH* genotypes and All-cancer risk in 100kGP
21	Associations between various germline *MUTYH* genotypes and All-cancer risk in UKB
22	Associations between bi-allelic germline *MUTYH* mutations and risk of individual cancer types in 100kGP
23	Associations between bi-allelic germline *MUTYH* mutations and risk of individual cancer types in UKB
24	Associations between mono-allelic germline *MUTYH* mutations and risk of various non-CRC cancers in 100kGP
25	Associations between mono-allelic germline *MUTYH* mutations and risk of various non-CRC cancers in UKB
26	Germline *de novo* mutations in offspring of carriers of mono-allelic *MUTYH* mutations.
27	Germline mono-allelic *MBD4* mutations, mutational processes in 100kGP cancers, and CRC risk.
**Supplementary Figures**	
1	C:G>A>T trinucleotide mutation channels in signatures SBS18 and SBS36
2	CRC-excluded patients used in germline and somatic molecular analyses
3	Selected somatic molecular features of tumours in the 100kGP All-cancer analysis in relation to germline *MUTYH* status

Throughout, group1: bi-allelic germline *MUTYH* mutations; group 2a: mono-allelic germline *MUTYH* mutation with LOH wildtype allele; group 2b: mono-allelic germline *MUTYH* mutation with LOH mutant allele; group 2c: mono-allelic germline *MUTYH* mutation with no LOH wildtype; group 3: germline *MUTYH* wildtype

Supplementary Table 1. Summary of somatic molecular features of 100kGP CRCs. μ mean, σ standard deviation, M median. SCVPM, somatic coding variants per Mb, a measure of tumour mutation burden focussed on variants more likely to have pathogenic effects. Group 1: bi-allelic germline *MUTYH* mutations; group 2a: mono-allelic germline *MUTYH* mutations with LOH of germline wildtype allele; group 2b: mono-allelic germline *MUTYH* mutations with LOH of germline mutant allele; group 2c: mono-allelic germline *MUTYH* mutations with no LOH; group 3: wildtype *MUTYH* alleles.

Molecular feature		Group 1	Group 2a	Group 2b	Group 2c	Group 3
	n	4	2	5	37	2465
Age	μ	52	71	68	67	68
Presence SBS36	μ	0.75	0.50	0.00	0.00	0.00
	σ	0.50	0.71	0.00	0.00	0.06
	M	1.00	0.50	0.00	0.00	0.00
Burden SBS36	μ	24366	5999	0	0	17
	σ	16290	8484	0	0	464
	M	32033	5999	0	0	0
Activity SBS36	μ	0.377	0.231	0.000	0.000	0.001
	σ	0.372	0.327	0.000	0.000	0.013
	M	0.327	0.231	0.000	0.000	0.000
Presence SBS18	μ	0.50	0.50	0.80	0.46	0.27
	σ	0.58	0.71	0.45	0.51	0.44
	M	0.50	0.50	1.00	0.00	0.00
Burden SBS18	μ	15914	2117	3461	2689	1146
	σ	20258	2994	2190	3610	2307
	M	10692	2117	3833	0	0
Activity SBS18	μ	0.171	0.131	0.176	0.115	0.054
	σ	0.201	0.195	0.101	0.134	0.093
	M	0.151	0.131	0.214	0.000	0.000
Burden C>A	μ	34464	8249	4035	6739	9542
	σ	6067	4846	1140	7330	44828
	M	34765	8249	4186	3980	3668
Activity C>A	μ	0.448	0.379	0.231	0.203	0.173
	σ	0.223	0.101	0.030	0.070	0.050
	M	0.448	0.379	0.218	0.193	0.172
SCVPM (TMB)	μ	36.4	5.2	3.4	12.33	17.49
	σ	32.7	1.9	1.6	25.65	36.33
	M	27.3	5.2	4.1	3.80	3.86

Supplementary Table 2. Summary of somatic molecular features of 100kGP MSS CRCs.
Legend as in **Supplementary Table 2**.

Molecular feature		Group 1	Group 2a	Group 2b	Group 2c	Group 3
	n	1	2	5	30	1875
Age	μ	53	71	68	69	67
Presence SBS36	μ	1	0.50	0	0	0.01
	σ	-	0.71	0	0	0.07
	M	1	0.50	0	0	0
Burden SBS36	μ	33299	5999	0	0	22
	σ	-	8484	0	0	532
	M	33299	5999	0	0	0
Activity SBS36	μ	0.854	0.231	0	0	0.001
	σ	-	0.327	0	0	0.015
	M	0.854	0.231	0	0	0
Presence SBS18	μ	0	0.50	0.80	0.53	0.34
	σ	-	0.71	0.45	0.51	0.47
	M	0	0.50	1.00	1.00	0
Burden SBS18	μ	0	2117	3461	3045	1397
	σ	-	2994	2190	3677	2265
	M	0	2117	3833	1997	0
Activity SBS18	μ	0	0.131	0.176	0.133	0.068
	σ	-	0.195	0.101	0.137	0.101
	M	0	0.131	0.214	0.162	0
Burden C>A	μ	26759	8249	4035	4657	3912
	σ	-	4846	1140	3781	12538
	M	26759	8249	4186	3760	3238
Activity C>A	μ	0.686	0.379	0.231	0.219	0.185
	σ	-	0.101	0.030	0.065	0.040
	M	0.686	0.379	0.218	0.217	0.182
SCVPM (TMB)	μ	8.5	5.2	3.4	3.9	3.7
	σ	-	1.9	1.6	2.4	3.3
	M	8.5	5.2	4.1	3.4	3.4

Supplementary Table 3. SBS36 & SBS18 presence & absence in germline MUTYH mutation groups in cases with CRC, 'All-Cancer', and 'CRC-excluded' from the 100kGP. The upper table shows tumour counts; the lower table shows associations with *MUTYH* groups (1=bi-allelic germline mutations, 2a=mono-allelic germline mutation + loss of the wildtype allele, 2b= mono-allelic germline mutation + loss of the mutant allele, 2c= mono-allelic germline mutation with no loss, 3=germline MUTYH-wildtype). ND=not determined.

Tumour type	Germline *MUTYH* group	SBS36 present	SBS36 absent	Total	SBS18 present	SBS18 absent	Total
CRC	1	3	1	4	2	2	4
	2a	1	1	2	1	1	2
	2b	0	5	5	4	1	5
	2c	0	37	37	17	20	37
	All mono-allelic	1	43	44	22	22	44
	3	10	2455	2465	669	1796	2465
	Total	14	2499	2513	693	1820	2513
All-cancer	1	3	2	5	3	2	5
	2a	5	7	12	3	9	12
	2b	0	14	14	5	9	14
	2c	3	242	245	46	199	245
	All mono-allelic	8	263	271	57	219	271
	3	18	14929	14947	1232	13715	14947
	Total	29	15194	15223	1289	13934	15223
CRC excluded	1	0	1	1	1	0	1
	2a	4	6	10	2	8	10
	2b	0	9	9	1	9	9
	2c	3	199	202	26	176	202
	All mono-allelic	7	214	221	29	192	221
	3	8	12304	12312	530	11782	12312
	Total	15	12519	12534	560	11974	12534

	Signature	MUTYH groups	Odds ratio	95% CI	P	Test
CRC	SBS36	1 *vs* 2a/b/c+3	681	65.7-7067	1.10×10^{-6}	Fisher's exact
		2a *vs* 2b/c	ND	ND	0.090	Fisher's exact
		2a/b/c *vs* 3	5.71	0.72-46	0.354	χ^2
	SBS18	1 *vs* 2a/b/c+3	2.63	0.37-19	0.612	Fisher's exact
		2a *vs* 2b/c	1	0.06-17	1	Fisher's exact
		2a/b/c *vs* 3	2.685	1.51-4.76	4.48×10^{-4}	χ^2
All-cancer	SBS36	1 *vs* 2a/b/c+3	141	141-5464	1.24×10^{-7}	Fisher's exact
		2a *vs* 2b/c	61	12.1-307	7.07×10^{-6}	Fisher's exact
		2a/b/c *vs* 3	25.2	10.9-59	$<10^{-7}$	χ^2
	SBS18	1 *vs* 2a/b/c+3	16.7	2.79-100	9.90×10^{-4}	Fisher's exact
		2a *vs* 2b/c	1.36	3.55-320	0.881	Fisher's exact
		2a/b/c *vs* 3	2.90	2.15-3.90	$<10^{-7}$	χ^2
CRC excluded	SBS36	1 *vs* 2a/b/c+3	1	ND	1	Fisher's exact
		2a *vs* 2b/c	46	8.42-254	1.42×10^{-4}	Fisher's exact
		2a/b/c *vs* 3	50	18.1-140	$<10^{-7}$	Fisher's exact
	SBS18	1 *vs* 2a/b/c+3	1	ND	0.089	Fisher's exact
		2a *vs* 2b/c	1.71	0.345-8.49	0.769	Fisher's exact
		2a/b/c *vs* 3	3.36	1.76-5.52	3.44×10^{-7}	χ^2

Supplementary Table 4. Pairwise associations in univariable analyses between germline MUTYH genotypes and cancer molecular features in 100kGP CRCs, All-cancer, and CRC-excluded tumour sets. For binary variables, ORs & 95% CIs are from 2x2 tables & P values from χ^2 or Fisher's exact tests, whereas for associations involving quantitative variables (burden & activity), P values are from Wilcoxon tests. Effect size metrics (i.e. βs (log(ORs)), standard errors (SE) & corresponding $P_{regress}$ values) are estimated from univariable regression with robust variances. ND, tests not performed owing to small sample numbers.

		CRC only				All-cancer				CRC-excluded			
Groups	Somatic features	P	β	SE	$P_{regress}$	P	β	SE	$P_{regress}$	P	β	SE	$P_{regress}$
1 *vs* 2+3	Presence SBS36	1.10x10^{-6}	0.746	0.034	<2.2x10^{-16}	1.24x10^{-7}	0.598	0.019	<2.2x10^{-16}	1	ND	ND	ND
	Burden SBS36	0.00080	24345	8145	0.00282	<2.2x10^{-16}	19487	7972	0.015	ND	ND	ND	ND
	Activity SBS36	0.0016	0.376	0.186	0.0434	<2.2x10^{-16}	0.301	0.163	0.064	ND	ND	ND	ND
	Presence SBS18	0.61	0.225	0.224	0.315	0.011	0.515	0.124	3.46x10^{-5}	0.089	ND	ND	ND
	Burden SBS18	0.11	14740	10129	0.150	1.47x10^{-5}	12764	8349	0.126	ND	ND	ND	ND
	Activity SBS18	0.12	0.116	0.100	0.250	1.24x10^{-5}	0.155	0.078	0.047	ND	ND	ND	ND
	Burden C>A	0.00080	24975	3161	4.08x10^{-15}	0.0026	21476	6958	2.03x10^{-3}	ND	ND	ND	ND
	Activity C>A	0.0016	0.275	0.112	0.014	0.0011	0.237	0.102	0.020	ND	ND	ND	ND
	TMB	<2.2x10^{-16}	19.0	16.37	0.245	0.025	21.3	14.5	0.143	ND	ND	ND	ND
2a *vs* 2b/c	Presence SBS36	0.091	ND	ND	ND	7.07x10^{-6}	0.405	0.044	<2.2x10^{-16}	1.42x10^{-4}	0.386	0.164	0.019
	Burden SBS36	7.70x10^{-6}	ND	ND	ND	2.87x10^{-16}	2134	1052	0.043	3.30x10^{-12}	1361	653	0.038
	Activity SBS36	7.70x10^{-6}	ND	ND	ND	2.87x10^{-16}	0.121	0.053	0.024	3.30x10^{-12}	0.099	0.052	0.057
	Presence SBS18	1.00	ND	ND	ND	0.881	0.053	0.118	0.654	0.77	0.072	0.135	0.594
	Burden SBS18	0.90	ND	ND	ND	0.569	289	710	0.684	0.51	593	772	0.443
	Activity SBS18	0.81	ND	ND	ND	0.558	0.020	0.034	0.569	0.52	0.024	0.034	0.473
	Burden C>A	0.25	ND	ND	ND	0.031	-2499	3536	0.480	0.040	-3335	4264	0.435
	Activity C>A	0.026	ND	ND	ND	0.0062	0.086	0.039	0.026	0.023	0.071	0.041	0.085
	TMB	0.38	ND	ND	ND	0.141	-3.48	1.87	0.064	0.185	-2.67	2.11	0.207
2 *vs* 3	Presence SBS36	0.35	0.187	0.010	0.063	<10^{-7}	0.028	0.003	<2.2x10^{-16}	<10^{-7}	0.031	0.012	0.009
	Burden SBS36	0.062	255.9	273	0.348	<2.2x10^{-16}	95.6	52.2	0.067	<2.2x10^{-16}	66.4	34.2	0.052
	Activity SBS36	0.062	0.0099	0.011	0.348	<2.2x10^{-16}	0.006	0.003	0.028	<2.2x10^{-16}	0.005	0.003	0.041
	Presence SBS18	4.48x10^{-4}	0.229	0.068	7.61x10^{-4}	<10^{-7}	0.117	0.017	6.98x10^{-12}	4.44x10^{-7}	0.088	0.023	1.13x10^{-4}
	Burden SBS18	9.59x10^{-5}	1605	517	1.99x10^{-3}	2.19x10^{-12}	735	176	2.98x10^{-5}	8.88x10^{-12}	462	143	1.26x10^{-3}
	Activity SBS18	2.61x10^{-5}	0.069	0.020	5.70x10^{-4}	1.18x10^{-12}	0.029	0.006	1.05x10^{-6}	8.60x10^{-12}	0.018	0.005	1.16x10^{-4}
	Burden C>A	0.58	-3041	1369	0.026	0.045	833	3207	0.795	0.016	1648	3922	0.674
	Activity C>A	5.19x10^{-5}	0.041	0.012	3.65x10^{-4}	1.22x10^{-6}	0.0180	0.005	3.78x10^{-4}	2.02x10^{-5}	0.012	0.005	0.027
	TMB	0.34	-6.50	3.64	0.075	0.811	-0.732	1.652	0.658	0.445	0.334	1.851	0.857

Supplementary Table 5. Multivariable regression analysis for associations between germline MUTYH genotypes and mutational processes in 100kGP CRCs. Generalised linear models (glm) were used for binary outcome variables and linear regression with robust variances for quantitative outcomes. Included as co-variables in each initial regression model were tumour location (proximal=between caecum and splenic flexure, distal=between descending colon and rectum), MSI, primary or metastasis, age, sex, tumour purity, prior chemotherapy, prior radiotherapy and TMB defined as somatic coding variants per Mb (unless an outcome). Non-significant variables at P=0.05 were sequentially excluded in reverse stepwise regression analysis until all variables with P<0.05 remained. Where the *MUTYH* group variable remained significantly associated with molecular features (P<0.05), other positively associated co-variables in the final model are shown. Where the *MUTYH* variable was excluded from the final model (P>0.05), NS is shown. The table complements **Table 2** and **Supplementary Tables 4 & 5**. **Supplementary Tables 17 & 19** provide equivalent data for All-cancer and CRC-excluded analyses.

Groups	Somatic features	β	SE	P	Positively associated co-variables, P<0.05
1 v 2+3	Presence SBS36	0.749	0.249	0.0027	MSS
	Burden SBS36	24345	8145	0.0028	
	Activity SBS36	0.377	0.186	0.043	MSS
	Presence SBS18			NS	
	Burden SBS18			NS	
	Activity SBS18			NS	
	Burden C>A			NS	
	Activity C>A	0.280	0.107	0.0090	Low TMB
	TMB			NS	
2 v 3	Presence SBS36			NS	
	Burden SBS36			NS	
	Activity SBS36			NS	
	Presence SBS18	0.244	0.075	9.27×10^{-4}	MSS, proximal location, primary, sex
	Burden SBS18	1741	511	6.80×10^{-4}	MSS, proximal location, primary
	Activity SBS18	0.081	0.022	2.22×10^{-4}	MSS, proximal location, primary
	Burden C>A	1639	833	0.049	MSS, proximal location
	Activity C>A	0.042	0.010	5.69×10^{-5}	MSS, proximal location, age
	TMB			NS	

Supplementary Table 6. Univariable associations between MUTYH genotype (group 2 vs *group 3) and (a) CRC location (proximal v distal colorectum) and (b) MSI.* Despite their presence as significant predictors in the multivariable models, proximal location and MSS status were not associated with group 2 in the univariable analyses *versus* group3.

		Proximal	Distal	Total		MSI	MSS	Total
MUTYH mono-allelic (group 2)	a	12	30	42	b	7	37	44
Wildtype (group 3)		979	1447	2426		569	1875	2444
Total		991	1477	2468		576	1912	2488

(a) OR=0.591, 95%CI 0.301-1.16; *P*=0.162, Fisher's exact test

(b) OR=0.623, 95%CI 0.276-1.406; *P*=0.332, Fisher's exact test

Supplementary Table 7. Associations between germline MUTYH genotypes and molecular features in 100kGP MSS CRC-only analyses. MSI+ and POL-mutant cancers were excluded. As per **Supplementary Table 5,** for binary variables, ORs and 95% CIs are from 2x2 tables and P values from χ^2 or Fisher's exact tests, whereas for associations involving quantitative variables (burden and activity), P values are from Wilcoxon tests. Effect size metrics (i.e. βs (log(ORs)), standard errors (SE) and corresponding $P_{regress}$ values) are estimated from univariable regression with robust variances. ND shows tests not performed owing to small sample numbers. Other annotation is as per **Supplementary Tables 4-6**. Group 2a: mono-allelic germline *MUTYH* mutation with LOH wildtype allele; group 2b: mono-allelic germline *MUTYH* mutation with LOH mutant allele; group 2c: mono-allelic germline *MUTYH* mutation with no LOH wildtype; group 3: germline *MUTYH* wildtype

(a) Univariable analyses

Groups	Somatic features	P	β	SE	$P_{regress}$
2a v 2b/c	Presence SBS36	0.11	ND	ND	ND
	Burden SBS36	4.83×10^{-6}	ND	ND	ND
	Activity SBS36	4.83×10^{-6}	ND	ND	ND
	Presence SBS18	1.00	ND	ND	ND
	Burden SBS18	0.78	ND	ND	ND
	Activity SBS18	0.94	ND	ND	ND
	Burden C>A	0.15	ND	ND	ND
	Activity C>A	0.029	ND	ND	ND
	TMB	0.24	ND	ND	ND
2 v 3	Presence SBS36	0.39	0.022	0.013	0.084
	Burden SBS36	0.083	302	325	0.352
	Activity SBS36	0.083	0.012	0.012	0.351
	Presence SBS18*	7.51×10^{-3}	0.230	0.083	5.82×10^{-3}
	Burden SBS18	4.65×10^{-4}	1654	566	3.51×10^{-3}
	Activity SBS18	1.13×10^{-4}	0.071	0.022	1.09×10^{-3}
	Burden C>A	0.046	856	661	0.196
	Activity C>A	1.30×10^{-5}	0.044	0.012	2.24×10^{-4}
	TMB	0.67	0.159	0.376	0.672

*OR=2.58, 95% CI 1.34-4.97, χ^2_1=8.53, P=7.51×10^{-3}

(b) Multiple regression analyses
Note that the borderline significant association in 'all CRC' analysis between group 2 and C>A burden (**Supplementary Table 5**) is not present in these MSS-only data.

Groups	Somatic features	B	SE	P	Positively associated co-variables, P<0.05
2 v 3	Presence SBS18	0.266	0.088	2.61×10^{-3}	Proximal location, primary CRC, sex
	Burden SBS18	2069	634	1.13×10^{-3}	Proximal location, primary CRC, higher TMB
	Activity SBS18	0.087	0.024	2.30×10^{-4}	Proximal location, primary CRC, female sex
	Activity C>A	0.045	0.011	4.61×10^{-5}	Proximal location, higher TMB, age

Supplementary Table 8. Comparison between SBS18-positive CRCs from mono-allelic MUTYH mutation carriers (n=17, group 2) and MUTYH-wildtype individuals (n=528, group 3). Median values are shown for quantitative variables, and numbers of tumours/total for binary variables (location, whole genome doubling, MSI, primary or metastasis). P values are derived from Wilcoxon tests or Fisher's exact tests. This analysis was performed on a sub-set of individuals from whom data such as whole genome doubling were available.

	Group 2	*Group 3*	*P*
SBS18 burden	5729	3859	0.0029
SBS18 activity	0.250	0.197	0.00019
C>A burden	5326	3904	0.0014
C>A activity	0.247	0.197	2.37×10^{-4}
SCVPM	4.20	3.71	0.120
Age (years)	71	70	0.803
Location (proximal colorectum)	11/17	263/528	0.336
Whole genome doubling	7/16	201/467	0.129
MSI	0/17	2/526	1.000
Primary	17/17	503/528	0.889

Supplementary Table 9. Associations between mono-allelic germline MUTYH mutations (group 2) and molecular features in 493 metastatic CRCs from Hartwig study. Fourteen CRCs were from germline mono-allelic mutation carriers. Data are from Fisher's exact test (for presence/absence) and from WIlcoxon tests (for all other measures). Median values are shown for burdens and activities, with means in brackets. Here, owing to the data available, TMB was expressed as the sum of all SBS mutations. MSS and MSI data were combined for this analysis, with all 23 MSI+ cancers in the *MUTYH*-wildtype group (hence the lower between TMB in group 2 cancers), and many cancers were sampled after receiving chemotherapy, which is likely to have introduced background mutational 'noise'. For these reasons, as for the 100kGP analyses, we regarded SBS18 as the most reliable measure of MUTYH function here, whereas measures of mutation burden were subject to additional factors of uncertain magnitude. We were reassured that most SBS18 measures, including burden, were associated with mono-allelic *MUTYH* carriers, as was C>A activity.

Groups	Somatic features	Group 2 v Group 3	P
2 *vs* 3	Presence SBS18	13/14 v 369/479	0.209
	Burden SBS18	2322 (2761) v 1582 (1678)	0.0061
	Activity SBS18	0.12 (0.15) v 0.06 (0.06)	$<2x10^{-5}$
	Burden C>A	3426 (3762) v 3496 (5374)	0.854
	Activity C>A	0.34 (0.41) v 0.24 (0.25)	$1x10^{-4}$
	TMB	17894 (19333) v 26652 (39179)	0.0002

Supplementary Table 10. Exome SNP array data for MUTYH p.Gly382Asp and Tyr179Cys. A total of 20 *MUTYH* variants, all of which were missense or possible splice changes and mostly predicted to be non-pathogenic, was present on these SNP arrays. p.Tyr179Cys and p.Gly396Asp were the only pathogenic variants found in our patients. Whilst a small number of homozygous p.Tyr179Cys and p.Gly396Asp carriers were found, there was no compound heterozygote p.Tyr179Cys/p.Gly396Asp individual. We cannot exclude, for example, that a small number (probably <1%) of the apparent mono-allelic p.Tyr179Cys or p.Gly396Asp mono-allelic mutants were actually compound heterozygotes with a second, rare, untyped pathogenic variant, and hence these data do not form part of the formal meta-analyses in **Table 1**.

	N mono-allelic cases/total	N mono-allelic controls/total	OR	95%CI	P
G396D/+	89/5554	90/8091	1.448	1.078-1.945	0.0174
Y165C/+	7/5574	14/8069	0.7235	0.292-1.793	0.640

Supplementary Table 11. Exploration of potential confounders that could have caused the association between mono-allelic MUTYH mutations and SBS18 burden in 100kGP CRCs. Results are from a multivariable linear model including only MSS, POL-proficient CRCs. SBS18 burden was the outcome and *MUTYH* groups 2 *versus* 3 (germline heterozygotes *versus* wildtype) were the main explanatory variable, with each co-variable included in turn. All models also included sex and age. Whilst some variables were significantly associated with SBS18 burden, the impact of the mono-allelic *MUTYH* genotype was still significant in all instances. Note that the association with ancestry was driven by a single individual of African descent. BER genes tested were *OGG1, UNG, SMUG1, TDG, NTHL1, MPG, NEIL1, NEIL2, NEIL3, PARP1, PARP2, PARP3, PARG* and *PARPBP.* Associations with age, sex and location were detected previously in other multivariable analyses (see above). Note that although not shown in the table, there was no association between SBS18 and measures of the microbiome, as previously reported by Cornish *et al*[27].

Co-variable	β	SE	P	Significance of *MUTYH* genotype after controlling for co-variable
Participant age	20.049	4.591	1.33×10^{-5}	1.86×10^{-5}
Participant sex	-259.060	108.71	0.017	1.61×10^{-5}
Prior chemotherapy or radiotherapy	-123.192	109.155	0.259	2.00×10^{-5}
Proximal vs distal colorectum location	1532.084	107.961	$<2e^{-16}$	4.15×10^{-7}
Tumour type - primary vs met	509.171	212.68	0.017	2.08×10^{-5}
Tumour stage (1-4)	-16.804	63.356	0.791	8.53×10^{-6}
Genetic ancestry – African vs European	1272.187	351.148	2.99×10^{-4}	1.91×10^{-5}
Genetic ancestry – American vs European	-884.907	328.171	0.007	1.91×10^{-5}
Genetic ancestry – East Asian vs European	-608.782	571.328	0.287	1.91×10^{-5}
Genetic ancestry – Mixed vs European	-13.181	165.507	0.937	1.91×10^{-5}
Tumour purity (ASCAT)	-10.157	238.683	0.970	2.06×10^{-5}
Tumour ploidy (ASCAT)	-59.761	63.045	0.343	2.73×10^{-5}
Presence of other BER mutation	87.909	468.086	0.851	2.15×10^{-5}

Supplementary Table 12. Geographical origins of the patients from different regions of England.

100kGP participants were recruited by one of 12 Genomic Medicine Centres. In order to create large enough sets of patients of some types, we reduced the number of locations to five by merging some geographically adjacent centres (e.g. London centres were combined). Centre identities are not provided for reasons of confidentiality. There was no association between *MUTYH* heterozygosity and location of the patient recruitment centre (heterogeneity χ^2, P=0.178).

Geographical location	No. *MUTYH* heterozygotes (group 2)	No. *MUTYH* wildtype (group 3)
1	9	517
2	6	509
3	13	798
4	7	393
5	9	237
Total	44	2454

Supplementary Table 13. Non-CRC cancers analysed for associations between MUTYH genotypes and somatic molecular features

**Tumour type**	**No.**
Bladder	408
Breast	2981
Childhood	165
Endocrine	30
Endometrial	924
Glioma	599
Haematological	825
Head & neck	282
Lung	1561
Melanoma	337
Ovarian	538
Prostate	574
Renal	1395
Sarcoma	1166
Testicular	59
Unknown primary	87
Upper gastrointestinal	600
Other	3
Total	12534

Supplementary Table 14. Summary of somatic molecular features of 100kGP All-cancer tumours. Legend as in **Supplementary Table 3**.

Molecular feature		Group 1	Group 2a	Group 2b	Group 2c	Group 3
	n	5	12	14	245	14947
Age	μ	48	55	68	62	62
Presence SBS36	μ	0.60	0.42	0	0.01	0
	σ	0.55	0.51	0	0.11	0.03
	M	1.00	0	0	0	0
Burden SBS36	μ	19493	2140	0	6	4
	σ	17826	3644	0	52	223
	M	30766	0	0	0	0
Activity SBS36	μ	0.301	0.122	0	0.001	0
	σ	0.364	0.184	0	0.009	0.007
	M	0.184	0	0	0	0
Presence SBS18	μ	0.60	0.25	0.36	0.19	0.08
	σ	0.55	0.45	0.50	0.39	0.28
	M	1.00	0	0	0	0
Burden SBS18	μ	13060	1295	1372	984	283
	σ	18670	2379	2082	2961	1184
	M	1641	0	0	0	0
Activity SBS18	μ	0.170	0.063	0.072	0.041	0.015
	σ	0.174	0.117	0.104	0.096	0.054
	M	0.167	0	0	0	0
Burden C>A	μ	27922	4875	3104	7618	6431
	σ	15542	4037	2335	55234	38834
	M	34294	4240	2408	1864	1671
Activity C>A	μ	0.394	0.257	0.183	0.170	0.157
	σ	0.228	0.132	0.051	0.079	0.075
	M	0.312	0.235	0.172	0.148	0.138
SCVPM (TMB)	μ	29.3	4.0	3.5	7.7	8.1
	σ	32.5	2.6	3.3	28.3	28.9
	M	19.2	3.3	3.1	2.2	2.2

Supplementary Table 15. Summary of somatic molecular features of 100kGP CRC-excluded tumours. Legend as in **Supplementary Table 2**.

Molecular feature		Group 1	Group 2a	Group 2b	Group 2c	Group 3
n		1	10	9	202	12312
Age	μ	31	52	68	61	61
Presence SBS36	μ	0	0.40	0	0.001	0
	σ	-	0.52	0	0.010	0.03
	M	0	0	0	0	0
Burden SBS36	μ	0	1368	0	7	2
	σ	-	2064	0	57	131
	M	0	0	0	0	0
Activity SBS36	μ	0	0.100	0	0.024	0
	σ	-	0.163	0	0.068	0.006
	M	0	0	0	0	0
Presence SBS18	μ	1	0.20	0.11	0.13	0.04
	σ	-	0.42	0.33	0.34	0.20
	M	1	0	0	0	0
Burden SBS18	μ	9818	1130	212	551	101
	σ	-	2396	636	2159	628
	M	9818	0	0	0	0
Activity SBS18	μ	0.167	0.048	0.014	0.024	0.007
	σ	-	0.106	0.044	0.068	0.036
	M	0.167	0	0	0	0
Burden C>A	μ	1756	4201	2586	7756	5736
	σ	-	3738	2715	60757	37178
	M	1756	3136	1636	1566	1422
Activity C>A	μ	0.179	0.233	0.156	0.162	0.153
	σ	-	0.128	0.040	0.077	0.079
	M	0.179	0.200	0.161	0.142	0.133
SCVPM (TMB)	μ	0.9	3.8	3.5	6.6	6.0
	σ	-	2.8	4.1	28.5	26.4
	M	0.9	2.7	2.6	1.9	1.8

Supplementary Table 16. Summary of somatic molecular features of Upper GI cancers. Legend as in **Supplementary Table 2**.

Molecular feature		Group 2c (all MSS)	Group 3 (all)	Group 3 (MSS only)
n		10	371	360
Age	μ	69	67	69
Presence SBS36	μ	0	0	0
	σ	0	0	0
	M	0	0	0
Burden SBS36	μ	0	0	0
	σ	0	0	0
	M	0	0	0
Activity SBS36	μ	0	0	0
	σ	0	0	0
	M	0	0	0
Presence SBS18	μ	0.30	0.18	0.19
	σ	0.48	0.39	0.40
	M	0	0	0
Burden SBS18	μ	2346	726	809
	σ	4476	1771	1943
	M	0	0	0
Activity SBS18	μ	0.078	0.031	0.032
	σ	0.132	0.070	0.071
	M	0	0	0
Burden C>A	μ	4287	2812	2527
	σ	3339	2712	1894
	M	2583	2136	2043
Activity C>A	μ	0.191	0.144	0.146
	σ	0.058	0.036	0.037
	M	0.192	0.139	0.140
SCVPM (TMB)	μ	3.5	4.7	2.9
	σ	2.5	11.0	3.2
	M	2.8	2.4	2.3

Supplementary Table 17. Multiple regression analysis of 100kGP All-cancer and CRC-excluded data sets. Incorporated co-variables were (1) origin from metastasis, (2) older age, (3) male sex, (4) lower tumour purity, (5) higher TMB/SCVPM (unless the outcome), (6) Prior radiotherapy, (7) Prior chemotherapy, (8) FFPE or PCR sequencing library. Note that CRC sequences were all from PCR-free libraries, and thus (8) was only used for All-cancer analysis. The three main tests – germline bi-allelic *MUTYH* mutation carriers versus others (group 1 *vs* groups 2+3), mono-allelic mutation carriers with somatic loss of the wildtype allele (Group 2a *vs* groups 2b/c) & mono-allelic carriers *vs* wildtype (group 2 *vs* group 3) – are shown. ND, not analysed owing to small sizes of some groups, NS, not significant in final regression model at nominal P<0.05. Columns "Other cov" show co-variables remaining in the final model at nominal P<0.05. If the direction of effect of the associated variable is opposite to the listed above, it is shown by a – sign.

1 *vs* 2+3	All-cancer				CRC-excluded			
Somatic features	β	SE	P	Other cov	β	SE	P	Other cov
Presence SBS36	0.599	0.245	0.015	-5,-8			ND	
Burden SBS36	19487	7972	0.015				ND	
Activity SBS36			NS				ND	
Presence SBS18	0.533	0.024	0.025	2,3,-5,-6,7,-8			ND	
Burden SBS18			NS				ND	
Activity SBS18	0.158	0.077	0.040	2,3,-5,-6,7,-5,-8			ND	
Burden C>A			NS				ND	
Activity C>A	0.236	0.103	0.022	2,3,-5,-6,7,-5,-8			ND	
TMB			NS				ND	

2a *vs* 2b/c	All-cancer				CRC-excluded			
Somatic features	β	SE	P	Other cov	β	SE	P	Other cov
Presence SBS36	0.405	0.007	6.90×10^{-3}		0.386	0.164	0.019	
Burden SBS36	2134	1052	0.043		1361	653	0.038	
Activity SBS36	0.121	0.053	0.024				NS	
Presence SBS18			NS				NS	
Burden SBS18			NS				NS	
Activity SBS18			NS				NS	
Burden C>A			NS				NS	
Activity C>A	0.096	0.014	3.63×10^{-3}	-1,2	0.084	0.034	0.013	-1,2,5
TMB			NS				NS	

2 *vs* 3	All-cancer				CRC-excluded			
Somatic features	β	SE	P	Other cov	β	SE	P	Other cov
Presence SBS36	0.028	0.010	0.006	-5,-8	0.031	0.012	8.59×10^{-3}	-5,-8
Burden SBS36			NS				NS	
Activity SBS36	6.06×10^{-3}	2.77×10^{-3}	0.028	-5,-8	5.47×10^{-3}	2.68×10^{-3}	0.041	-8
Presence SBS18	0.116	0.024	1.56×10^{-6}	2,-5,-8	0.088	0.023	1.19×10^{-4}	-1,2,-3,-5,-8
Burden SBS18	732	175	2.99×10^{-5}	2,-5,-8	462	177143	1.22×10^{-3}	-1,2,3,-5,7,-8
Activity SBS18	0.029	0.006	9.68×10^{-7}	2,-5,-8	0.018	0.005	1.08×10^{-5}	-1,2,-5,-8
Burden C>A			NS				NS	
Activity C>A	0.017	0.005	6.84×10^{-4}	-1,2,4,5,-6,7,-8	0.010	0.005	0.042	-1,2,4,5,-6,7,-8
TMB			NS				NS	

Supplementary Table 18. Pairwise associations in univariable analyses between germline MUTYH genotypes and molecular features in upper gastrointestinal, breast, prostate and endometrial cancers from 100kGP. Upper GI cancers included, cholangiocarcinomas (n=33), gastric carcinomas (n=81), pancreatic carcinomas (n=125), hepatocellular carcinomas (n=24), oesophageal adenocarcinomas (n=96), small intestinal adenocarcarcinomas (n=8) and others (n=16). For binary variables, P are values from χ^2 or Fisher's exact tests, whereas for associations involving quantitative variables (burden and activity), P values are from Wilcoxon tests. Effect size metrics (i.e. βs (log(ORs)), standard errors (SE) and corresponding $P_{regress}$ values) are estimated from univariable regression with robust variances. ND shows tests not performed owing to small sample numbers in one or more groups. No bi-allelic germline mutation carriers were present in any of the three cancer types. No prostate cancer showed LOH at the *MUTYH* locus. No upper GI cancer was from group 2a or 2b. SBS36 was very rare (<0.2% presence) or absent in all four cancer types. SBS18 was present in 19% upper GI, 4% breast, 1% prostate and 17% endometrial cancers. It is likely that the low SBS18 prevalence in breast and prostate tumours, relatively small sample size in upper GI, prostate and endometrial cancers, and heterogeneity of upper GI cancer types all hampered the power of the statistical tests, especially the regression analyses, given that the frequency of mono-allelic mutation carriers is only ~2.0%. The non-parametric and categorical tests are, however, consistent with positive effects of loss of the germline wildtype allele (group 2a) and of the mono-allelic germline genotype (group 2) on SBS36 and SBS18 respectively.

MUTYH		Upper GI cancer (n=383)				Breast cancer (n=2,981)				Prostate cancer (n=574)				Endometrial cancer (n=835)			
Groups	Somatic features	P	β	SE	$P_{regress}$	P	β	SE	$P_{regress}$	P	β	SE	$P_{regress}$	P	β	SE	$P_{regress}$
2a *vs* 2b/c	Presence SBS36	ND	ND	ND	ND	0.083	ND	ND	ND	ND	ND	ND	ND	0.11	ND	ND	ND
	Burden SBS36	ND	ND	ND	ND	$2.71x10^{-6}$	ND	ND	ND	ND	ND	ND	ND	$6.15x10^{-5}$	ND	ND	ND
	Activity SBS36	ND	ND	ND	ND	$2.71x10^{-6}$	ND	ND	ND	ND	ND	ND	ND	$6.15x10^{-5}$	ND	ND	ND
	Presence SBS18	ND	ND	ND	ND	1	ND	ND	ND	ND	ND	ND	ND	1	ND	ND	ND
	Burden SBS18	ND	ND	ND	ND	0.62	ND	ND	ND	ND	ND	ND	ND	0.70	ND	ND	ND
	Activity SBS18	ND	ND	ND	ND	0.62	ND	ND	ND	ND	ND	ND	ND	0.70	ND	ND	ND
	Burden C>A	ND	ND	ND	ND	0.13	ND	ND	ND	ND	ND	ND	ND	0.74	ND	ND	ND
	Activity C>A	ND	ND	ND	ND	0.032	ND	ND	ND	ND	ND	ND	ND	0.21	ND	ND	ND
	TMB	ND	ND	ND	ND	0.52	ND	ND	ND	ND	ND	ND	ND	0.78	ND	ND	ND
2 *vs* 3	Presence SBS36	ND	ND	ND	ND	0.19	0.019	0.021	0.360	0.035	ND	ND	ND	0.046	ND	ND	ND
	Burden SBS36	ND	ND	ND	ND	0.003	0.113	0.115	0.326	$6.55x10^{-14}$	ND	ND	ND	$5.93x10^{-11}$	ND	ND	ND
	Activity SBS36	ND	ND	ND	ND	0.003	0.010	0.010	0.326	$6.55x10^{-14}$	ND	ND	ND	$5.93x10^{-11}$	ND	ND	ND
	Presence SBS18	0.535	0.183	0.020	0.449	0.013	0.091	0.048	0.061	0.003	0.196	0.133	0.141	0.55	0.071	0.097	0.465
	Burden SBS18	0.260	1620	1418	0.254	$7.06x10^{-4}$	169	95	0.076	$1.47x10^{-13}$	134	93	0.151	0.38	101	153	0.511
	Activity SBS18	0.212	0.046	0.042	0.266	$6.56x10^{-4}$	0.019	0.010	0.060	$1.47x10^{-13}$	0.023	0.015	0.143	0.33	0.016	0.018	0.376
	Burden C>A	0.195	1474	1065	0.167	0.18	46	143	0.745	0.55	-589	249	0.018	0.60	18851	45200	0.677
	Activity C>A	0.003	0.048	0.018	0.010	0.001	0.021	0.008	0.009	0.18	0.008	0.007	0.201	0.067	0.024	0.022	0.260
	TMB	0.545	-1.25	0.978	0.202	0.86	-0.49	0.27	0.072	0.37	-0.60	0.246	0.016	0.89	8.04	20.1	0.690

Supplementary Table 19. Multiple regression analysis of Upper GI cancers.

Incorporated co-variables were (1) origin from metastasis, (2) older age, (3) male sex, (4) lower tumour purity, (5) higher TMB/SCVPM (unless the outcome), (6) Prior radiotherapy, (7) Prior chemotherapy, (8) FFPE or PCR sequencing library, (9) MSI. Legend is otherwise as per **Supplementary Table 16**. If the direction of effect of the associated variable is opposite to the listed above, it is shown by a – sign.

2 *vs* 3	Upper GI cancers			
Somatic features	β	SE	P	Other cov
Presence SBS18	0.116	0.024	NS	
Burden SBS18	732	175	NS	
Activity SBS18	0.029	0.006	NS	
Burden C>A	1885	827	0.023	3, 5, -4
Activity C>A	0.051	0.018	0.004	1, 9, -4
TMB			NS	

Supplementary Table 20. Associations between germline MUTYH genotypes and All-cancer risk in 100kGP. Two-tailed Fisher's exact tests. To assess the overall cancer risk associated with germline *MUTYH* mutations, we studied 11,318 unrelated participants of European genetic ancestry with a malignant neoplasm in the 100kGP cancer domain. There was no specific enrichment of cases with colorectal polyposis. Germline bi-allelic mutation of *MUTYH* was very rare (frequency <0.05%), principally occurring in participants with colorectal cancer (compared with the control data set, OR=6.0, 95%CI 0.43-82.57, P=0.10), but occasionally present in other cancer cases. Considering all tumour types together, there was no significant enrichment of bi-allelic germline *MUTYH* mutations in cancer cases compared to the set of cancer-free controls (OR=1.90, 95%CI 0.22-22.77; P=0.66, P_{adj}=1.00).

Germline *MUTYH*	OR (95% CI)	P	Padj
Bi-allelic mutation	1.90 (0.22-22.77)	0.66	1.00
Mono-allelic mutation	1.01 (0.83-1.21)	0.96	1.00
Mono-allelic p.Gly396Asp	0.92 (0.74-1.15)	0.47	1.00
Mono-allelic p.Tyr179Cys	1.41 (0.92-2.14)	0.10	0.50
Mono-allelic PTV	1.09 (0.30-3.78)	1.00	1.00

Supplementary Table 21. Associations between various germline MUTYH genotypes and All-cancer risk in UKB. Two-tailed Fisher's exact tests. We used WES data from 12,694 unrelated cases of European genetic ancestry with a malignant neoplasm and 28,939 European unrelated, cancer-free controls from UKBiobank. Considering all tumour types together, there was a significant enrichment of bi-allelic germline *MUTYH* mutations in cancer cases compared to the set of controls (OR=15.97, 95%CI 2.05-717; P=1.44x10^{-3}). However, this was largely driven by six cases (0.05%) with CRC, and one control (0.004%) with presumed bi-allelic germline *MUTYH* mutations, driving a significant overall association with cancer risk (see also **Supplementary Table 13**).

Germline MUTYH	OR (95% CI)	P	Padj
Bi-allelic mutation	15.97 (2.05-717.19)	1.44×10^{-3}	0.01
Mono-allelic mutation	1.01 (0.86-1.18)	0.90	0.93
Mono-allelic p.Gly396Asp	1.02 (0.84-1.24)	0.85	0.93
Mono-allelic p.Tyr179Cys	0.97 (0.69-1.36)	0.93	0.93
Mono-allelic PTV	1.74 (0.78-3.82)	0.16	0.40

Supplementary Table 22. Associations between bi-allelic germline MUTYH mutations and risk of individual cancer types in 100kGP. Two-tailed Fisher's exact tests. We did not find significantly elevated levels of germline bi-allelic mutations in any of the individual cancer types before or after correcting for multiple testing. Note that the numbers of cases in 100kGP data may not be shown exactly if they lie between 1 and 5, for reasons of patient confidentiality. Germline bi-allelic loss of *MUTYH* has specifically been suggested to predispose to gynaecological cancers (including ovarian and endometrial) and breast cancers (Paller et al., 2024; Win et al., 2016), but we identified no participants with these cancer types who had germline bi-allelic *MUTYH* inactivation. Note that association statistics here are unadjusted.

Cancer type	Bi-allelic mutation	Total	OR (95% CI)	P	Padj
Bones & joints	0	227	0 (0-334.51)	1.00	1.00
Brain & other nervous system	0	514	0 (0-148.31)	1.00	1.00
Breast	0	2463	0 (0-31.03)	1.00	1.00
Colon & rectum	<5	2395	6.0 (0.43-82.57)	0.10	1.00
Endocrine system	0	72	0 (0-1031.6)	1.00	1.00
Eye & orbit	0	18	0 (0-4588.58)	1.00	1.00
Female genital system	0	1258	0 (0-61.23)	1.00	1.00
Leukaemia	0	307	0 (0-245.47)	1.00	1.00
Liver & intrahepatic bile duct	0	289	0 (0-264.51)	1.00	1.00
Lymphoma	0	114	0 (0-663.45)	1.00	1.00
Male genital system	<5	794	9.08 (0.15-174)	0.15	1.00
Mesothelioma	0	<5	0 (0-Inf)	1.00	1.00
Miscellaneous	0	91	0 (0-842.02)	1.00	1.00
Myeloma	0	99	0 (0-799.37)	1.00	1.00
Oral cavity & pharynx	0	232	0 (0-334.51)	1.00	1.00
Respiratory system	0	1362	0 (0-56.12)	1.00	1.00
Skin (excluding BCC & SCC)	0	1205	0 (0-63.35)	1.00	1.00
Soft tissue including heart	0	513	0 (0-147.72)	1.00	1.00
Upper gastrointestinal	0	314	0 (0-246.27)	1.00	1.00
Urinary system	0	1603	0 (0-47.64)	1.00	1.00

Supplementary Table 23. Associations between bi-allelic germline MUTYH mutations and risk of individual cancer types in UKB. Two-tailed Fisher's exact tests. With the exception of CRC, there was no evidence that bi-allelic germline *MUYTH* carriers were at increased risk of any specific cancer type. Note that association statistics here are unadjusted.

Cancer type	Bi-allelic mutation	Total	OR	P	Padj
Bones & joints	0	21	0 [0-Inf]	1.00	1.00
Brain & other nervous system	0	109	0 [0-8772.74]	1.00	1.00
Breast	1	2763	10.49 [0.13-817.67]	0.17	1.00
Colon & rectum	6	1067	164.37 [19.9-7159]	0.00	0.00
Endocrine system	0	124	0 [0-8129.18]	1.00	1.00
Eye & orbit	0	39	0 [0-16384]	1.00	1.00
Female genital system	0	819	0 [0-1349.21]	1.00	1.00
Leukaemia	0	220	0 [0-4729.22]	1.00	1.00
Liver & intrahepatic bile duct	0	225	0 [0-4649.84]	1.00	1.00
Lymphoma	1	519	56.27 [0.72-4229]	0.03	0.35
Male genital system	0	1794	0 [0-620]	1.00	1.00
Mesothelioma	0	40	0 [0-16384]	1.00	1.00
Miscellaneous	0	14	0 [0-Inf]	1.00	1.00
Myeloma	0	121	0 [0-8011.63]	1.00	1.00
Oral cavity & pharynx	0	183	0 [0-5559.68]	1.00	1.00
Respiratory system	0	452	0 [0-2396.91]	1.00	1.00
Skin (excluding BCC & SCC)	1	4506	6.41 [0.08-501.42]	0.25	1.00
Soft tissue including heart	0	109	0 [0-8986.17]	1.00	1.00
Upper gastrointestinal	0	264	0 [0-4039.61]	1.00	1.00
Urinary system	0	460	0 [0-2376.35]	1.00	1.00

Supplementary Table 24. Associations between mono-allelic germline MUTYH mutations and risk of various non-CRC cancers in 100kGP. Results are from two-tailed Fisher's exact tests. Five cancer (sub-) types studied by Barreiro et al are shown separately, indicated by *. Mono-allelic pathogenic germline *MUTYH* mutations (group 2) were present in 211 (1.80%) cases, a very similar frequency to that in controls (OR=1.01, 95% CI 0.83-1.21; *P*=0.96, *Padj*=1.00). Mono-allelic mutations were generally no more common in any of the other 19 ICD cancer types compared to controls, albeit with limited evidence of an increased risk of gynaecological cancer (OR=1.47, 95%CI 0.99-2.12; *P*=0.04, P_{adj}=0.58). [+] exact counts available through 100kGP. Note that association statistics here are unadjusted.

Cancer type	Mono-allelic mutation [+]	Total	OR [95% CI]	P	Padj
Bones & joints	<5	227	0.47 [0.06-1.74]	0.45	1.00
Brain & other nervous system	8	514	0.84 [0.36-1.69]	0.74	1.00
Breast	45	2463	0.98 [0.70-1.36]	1.00	1.00
Endocrine system	<5	72	0.75 [0.02-4.33]	1.00	1.00
Eye & orbit	0	18	0 [0-12.11]	1.00	1.00
Female genital system	34	1258	1.47 [0.99-2.12]	0.04	0.58
Leukaemia	<5	307	0.17 [0-0.98]	0.05	0.58
Liver & intrahepatic bile duct	5	289	0.93 [0.30-2.23]	1.00	1.00
Lymphoma	<5	114	0.47 [0.01-2.69]	0.73	1.00
Male genital system	18	794	1.23 [0.71-1.99]	0.42	1.00
Mesothelioma	0	<5	0 [0-1999.47]	1.00	1.00
Miscellaneous	<5	91	0.59 [0.01-3.39]	1.00	1.00
Myeloma	<5	99	2.23 [0.59-5.96]	0.11	0.73
Oral cavity & pharynx	7	232	1.65 [0.65-3.50]	0.21	0.73
Respiratory system	25	1362	0.99 [0.63-1.50]	1.00	1.00
Skin (excluding BCC & SCC)	22	1205	0.98 [0.60-1.53]	1.00	1.00
Soft tissue including heart	5	513	0.52 [0.17-1.24]	0.18	0.73
Upper gastrointestinal	9	314	1.56 [0.70-3.05]	0.20	0.73
Urinary system	30	1603	1.01 [0.67-1.48]	0.92	1.00
Oesophageal adenoca. *	0	18	0 [0-12.11]	1.00	1.00
Prostate adenocarcinoma *	10	410	1.32 [0.62-2.50]	0.35	1.00
Renal clear cell *	12	607	1.07 [0.54-1.91]	0.76	1.00
Sarcoma *	8	742	0.58 [0.25-1.16]	0.16	0.73

Supplementary Table 25. Associations between mono-allelic germline MUTYH mutations and risk of various non-CRC cancers in UKB. Two-tailed Fisher's exact tests. Five cancer (sub-)types studied by Barreiro et al are shown separately, indicated by *. Mono-allelic pathogenic germline *MUTYH* mutations (group 2) were present in 239 (1.88%) UKB cases, a very similar frequency to that in controls (OR=0.99, 95% CI 0.84-1.15; *P*=0.88, *Padj*=0.93). Overall, the frequency of mono-allelic mutations was not significantly elevated in any of the 20 cancer types compared to the controls in UKB, including gynaecological cancers. Note that association statistics here are unadjusted.

Cancer type	Mono-allelic mutation	Total	OR	P	Padj
Bones & joints	1	21	2.57 [0.06-16.09]	0.33	0.66
Brain & other nervous system	0	109	0 [0-1.77]	0.28	0.66
Breast	55	2763	1.04 [0.77-1.38]	0.77	1.00
Endocrine system	6	124	2.61 [0.93-5.89]	0.03	0.55
Eye & orbit	0	39	0 [0-5.11]	1.00	1.00
Female genital system	17	819	1.09 [0.63-1.77]	0.70	0.97
Leukaemia	3	220	0.71 [0.14-2.11]	0.80	1.00
Liver & intrahepatic bile duct	4	225	0.93 [0.25-2.43]	1.00	1.00
Lymphoma	13	519	1.32 [0.69-2.3]	0.33	0.66
Male genital system	24	1794	0.70 [0.44-1.05]	0.09	0.55
Mesothelioma	1	40	1.32 [0.03-7.80]	0.54	0.79
Miscellaneous	0	14	0 [0-15.51]	1.00	1.00
Myeloma	0	121	0 [0-1.59]	0.18	0.60
Oral cavity & pharynx	1	183	0.28 [0.01-1.60]	0.27	0.66
Respiratory system	8	452	0.92 [0.39-1.85]	1.00	1.00
Skin excluding BCC & SCC	77	4506	0.89 [0.69-1.14]	0.38	0.66
Soft tissue including heart	3	109	1.45 [0.29-4.38]	0.47	0.73
Upper gastrointestinal	7	264	1.40 [0.55-2.94]	0.36	0.66
Urinary system	11	460	1.26 [0.62-2.29]	0.39	0.66
Adrenocortical carcinoma *	0	2	0 [0-273.63]	1.00	1.00
Oesophageal *	5	135	1.97 [0.63-4.75]	0.19	0.60
Prostate *	21	1634	0.67 [0.41-1.04]	0.07	0.55
Renal *	8	215	1.98 [0.84-4.01]	0.07	0.55
Sarcoma *	3	66	2.44 [0.49-7.51]	0.13	0.58

Supplementary Table 26. Germline de novo mutations in offspring of carriers of mono-allelic MUTYH mutations. The table entries are the numbers of *de novo* mutations (DNMs) identified by 100kGP in offspring for which a single parent is a monby 100kGP.o-allelic *MUTYH* mutation carrier compared offspring who have a single parent who is a carrier of bi-allelic germline *MUTYH* mutations, and offspring whose parents have no germline *MUTYH* mutation. Data from stringent and non-stringent DNM calling pipelines are provided, as defined by 100kGP (see link in **Methods**). $P_{stringent}$ mono-allelic *v* wildtype = 0.55, t test; $P_{non\text{-}stringent}$ mono-allelic *v* wildtype = 0.016, t test). Stringent calls are recommended for use by 100,000 Genomes.

	Non-stringent			Stringent		
MUTYH genotype	Mean	SD	Median	Mean	SD	Median
Bi-allelic (n=2)	910	134	910	63	17	63
Mono-allelic (n=206)	1033	548	948	69	15	69
Wildtype (n=11,308)	980	306	915	70	24	69

Supplementary Table 27. Germline mono-allelic MBD4 mutations, mutational processes in 100kGP cancers, and CRC risk.

(a) Summary of P values for tests of association between mono-allelic *MBD4* mutation carriers and each of the four measures of C>T mutation in 100kGP cancers. Owing to limited data, and hence some uncertainty, regarding the effects of missense *MBD4* mutations, only protein-truncating mutations (stop-gained, frameshift or pathogenic splice, supported by Splice AI and Clinvar) were regarded as pathogenic. Owing to the presence of a short repeat in *MBD4* that is prone to background slippage in MSI+ cancers, the analyses were undertaken in MSS cancers only. In all instances with a significant association, the *MBD4* carriers showed higher measures than the wildtype comparison group. Single variable analyses used Wilcoxon test. Multivariable analysis used linear regression with robust variances, incorporating age, sex, tumour purity, SCVPM and prior therapy as co-variables. The results suggest, plausibly, that CRC-only analyses are underpowered relative to the other, larger data sets.

Cancer group	Statistical analysis	SBS1 burden	SBS1 activity	C>T burden	C>T activity
CRC	Univariable	>0.05	>0.05	>0.05	0.0810
N=2,039 (8 carriers)	Multivariable	>0.05	>0.05	>0.05	>0.05
CRC-excluded	Univariable	0.0132	0.00584	>0.05	>0.05
N=12,172 (6 carriers)	Multivariable	>0.05	>0.05	>0.05	>0.05
All cancer	Univariable	2.73×10^{-5}	3.95×10^{-5}	>0.05	0.0175
N=14,211 (14 carriers)	Multivariable	1.43×10^{-2}	9.55×10^{-3}	2.01×10^{-2}	>0.05

(b) Association between mono-allelic germline *MBD4* mutation and CRC risk in 100kGP and UKB.

.

Study	N mono-allelic cases/total	N mono-allelic controls/total	OR	95%CI	P
100kGP + CORGI	9/2,985	15/14,441	2.908	1.272-6.652	0.028
UK Biobank WES	1/1,224	7/9,599	1.121	0.138-9.121	1.000
UK Biobank WGS	10/4,834	281/142,813	1.051	0.559-1.977	0.966
Meta-analysis			1.75	1.09-2.81	0.020

The forest plot shows meta-analysis results with inverse variance weighting. P_{het}=0.032, I^2=71.0%

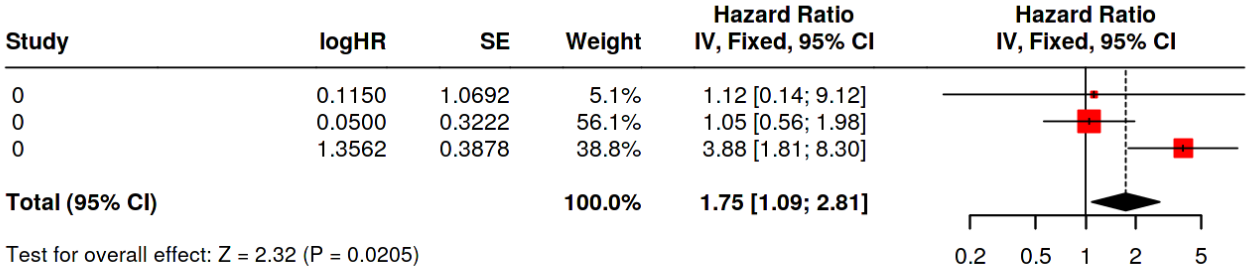

Supplementary Figure 1. C:G>A>T trinucleotide mutation channels in signatures SBS18 and SBS36. The activities of each of the 96 channels in the two COSMIC signatures are shown (https://cancer.sanger.ac.uk/signatures/sbs/sbs18/; https://cancer.sanger.ac.uk/signatures/sbs/sbs36/).

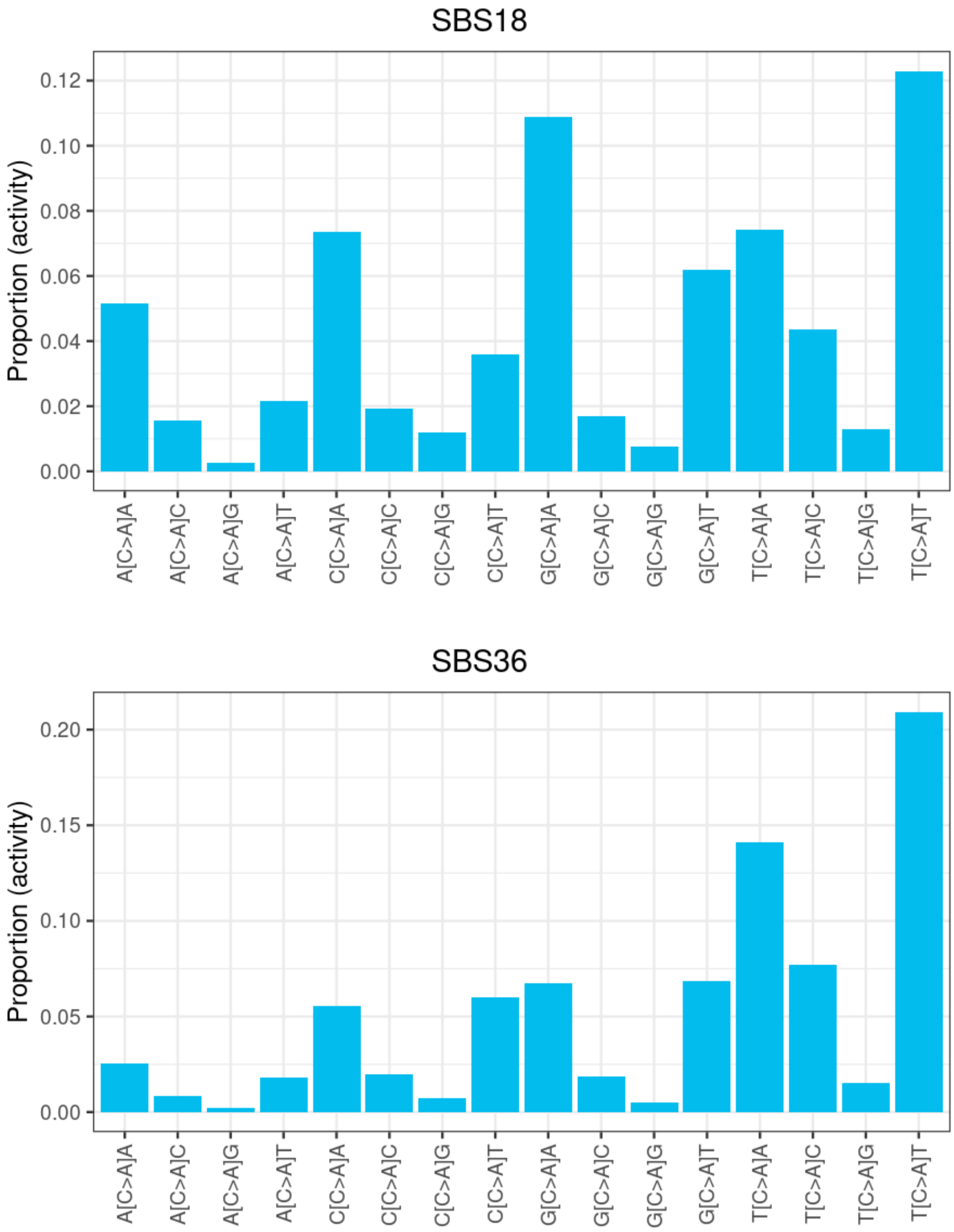

Supplementary Figure 2. CRC-excluded patients used in germline and somatic molecular analyses, sub-divided by germline MUTYH genotype, second hits at MUTYH and showing possible consequences for SBS signatures and C:G>A:T mutations. Note that these numbers may differ a little from those in other figures and table, owing to occasional patients or canecrs with missing data. The numbers of cancers may also exceed those of cases and controls, largely because ancestry and relatedness filtering were not applied to the latter.

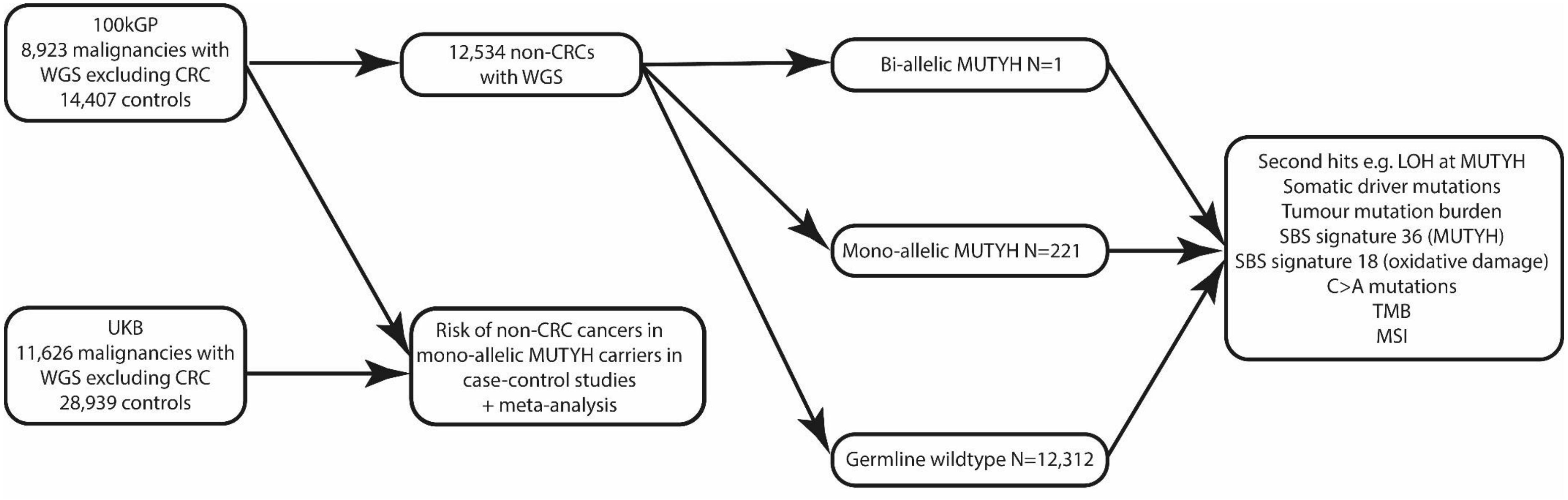

Supplementary Figure 3. Selected somatic molecular features of tumours in the 100kGP All-cancer analysis in relation to germline MUTYH status. (a) SBS36 proportional activity, (b) SBS18 proportion activity, (c) C:G>A:T burden, (d) C:G>A:T proportional activity and (e) tumour mutational burden (measured as SCVPM) in germline MUTYH genotype groups 1, 2a, 2b, 2c and 3. Group 1: bi-allelic germline MUTYH mutations; group 2a: mono-allelic germline MUTYH mutation with LOH wildtype allele; group 2b: mono-allelic germline MUTYH mutation with LOH mutant allele; group 2c: mono-allelic germline MUTYH mutation with no LOH wildtype; group 3: germline MUTYH wildtype. Log scales are used to aid display in some cases.

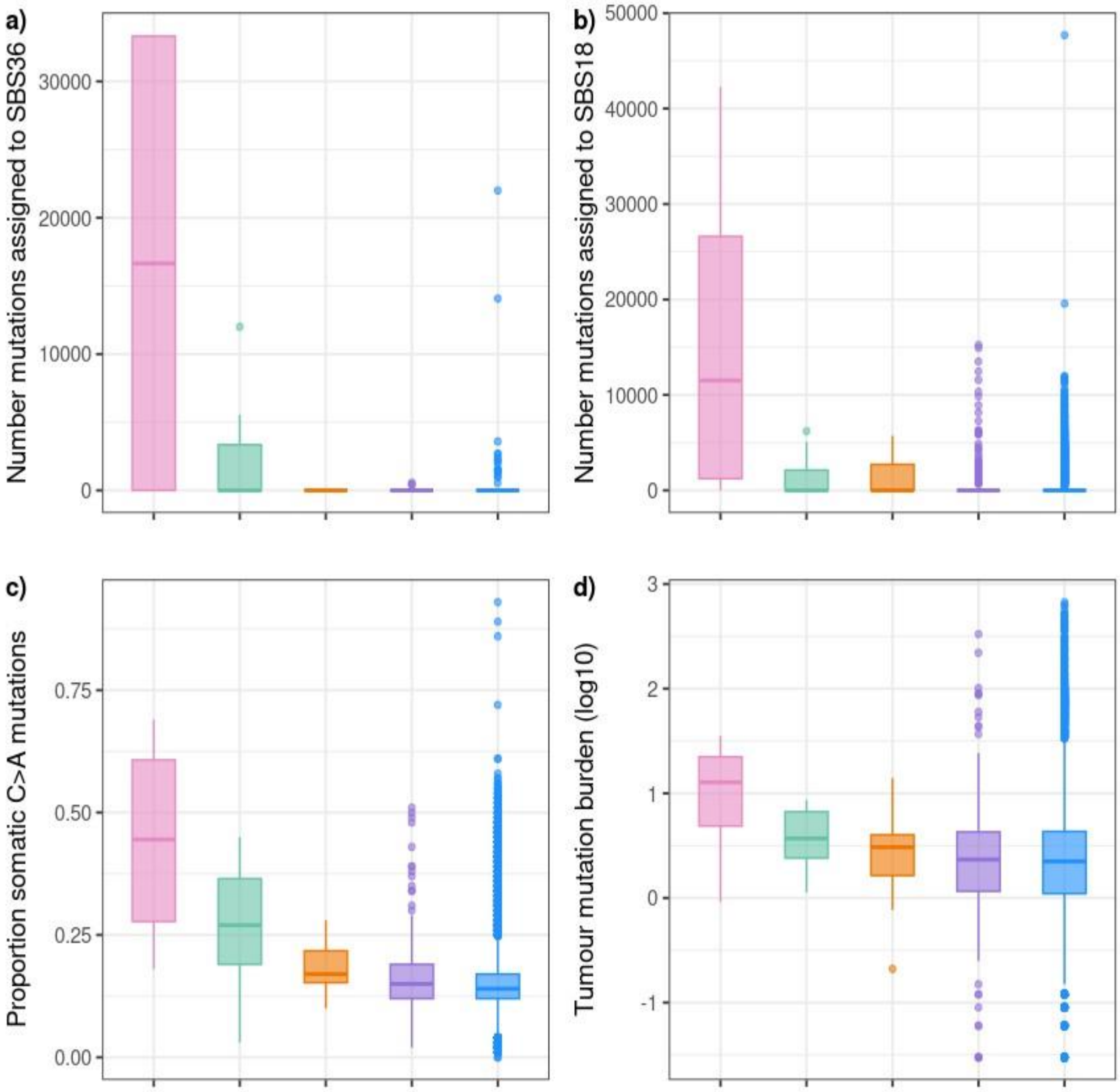

Data and association statistics corresponding to the above are shown in **Table 1** and **Supplementary Tables 13-18.**
